# Profiling the compendium of changes in *Saccharomyces cerevisiae* due to mutations that alter availability of the main methyl donor S-Adenosylmethionine

**DOI:** 10.1093/g3journal/jkae002

**Published:** 2024-01-07

**Authors:** McKayla Remines, Makailyn G Schoonover, Zoey Knox, Kailee Kenwright, Kellyn M Hoffert, Amila Coric, James Mead, Joseph Ampfer, Serigne Seye, Erin D Strome

**Affiliations:** Department of Biological Sciences, Northern Kentucky University, Highland Heights, KY 41099, USA; Department of Biological Sciences, Northern Kentucky University, Highland Heights, KY 41099, USA; Department of Biological Sciences, Northern Kentucky University, Highland Heights, KY 41099, USA; Department of Biological Sciences, Northern Kentucky University, Highland Heights, KY 41099, USA; Department of Biological Sciences, Northern Kentucky University, Highland Heights, KY 41099, USA; Department of Biological Sciences, Northern Kentucky University, Highland Heights, KY 41099, USA; Department of Biological Sciences, Northern Kentucky University, Highland Heights, KY 41099, USA; Department of Biological Sciences, Northern Kentucky University, Highland Heights, KY 41099, USA; Department of Biological Sciences, Northern Kentucky University, Highland Heights, KY 41099, USA; Department of Biological Sciences, Northern Kentucky University, Highland Heights, KY 41099, USA

**Keywords:** S-Adenosylmethionine, *SAM1*, *SAM2*, methyl cycle, Phenotypic Microarray, dNTPs, glutathione, arginine metabolism, tamoxifen, fluconazole, ergosterol biosynthesis, cisplatin

## Abstract

The *SAM1* and *SAM2* genes encode for S-Adenosylmethionine (AdoMet) synthetase enzymes, with AdoMet serving as the main cellular methyl donor. We have previously shown that independent deletion of these genes alters chromosome stability and AdoMet concentrations in opposite ways in *Saccharomyces cerevisiae*. To characterize other changes occurring in these mutants, we grew wildtype, *sam1Δ*/sam1*Δ*, and *sam2Δ*/sam2*Δ* strains in 15 different Phenotypic Microarray plates with different components and measured growth variations. RNA-Sequencing was also carried out on these strains and differential gene expression determined for each mutant. We explored how the phenotypic growth differences are linked to the altered gene expression, and hypothesize mechanisms by which loss of the *SAM* genes and subsequent AdoMet level changes, impact pathways and processes. We present 6 stories, discussing changes in sensitivity or resistance to azoles, cisplatin, oxidative stress, arginine biosynthesis perturbations, DNA synthesis inhibitors, and tamoxifen, to demonstrate the power of this novel methodology to broadly profile changes due to gene mutations. The large number of conditions that result in altered growth, as well as the large number of differentially expressed genes with wide-ranging functionality, speaks to the broad array of impacts that altering methyl donor abundance can impart. Our findings demonstrate that some cellular changes are directly related to AdoMet-dependent methyltransferases and AdoMet availability, some are directly linked to the methyl cycle and its role in production of several important cellular components, and others reveal impacts of *SAM* gene mutations on previously unconnected pathways.

## Introduction

**Fig. 1.**
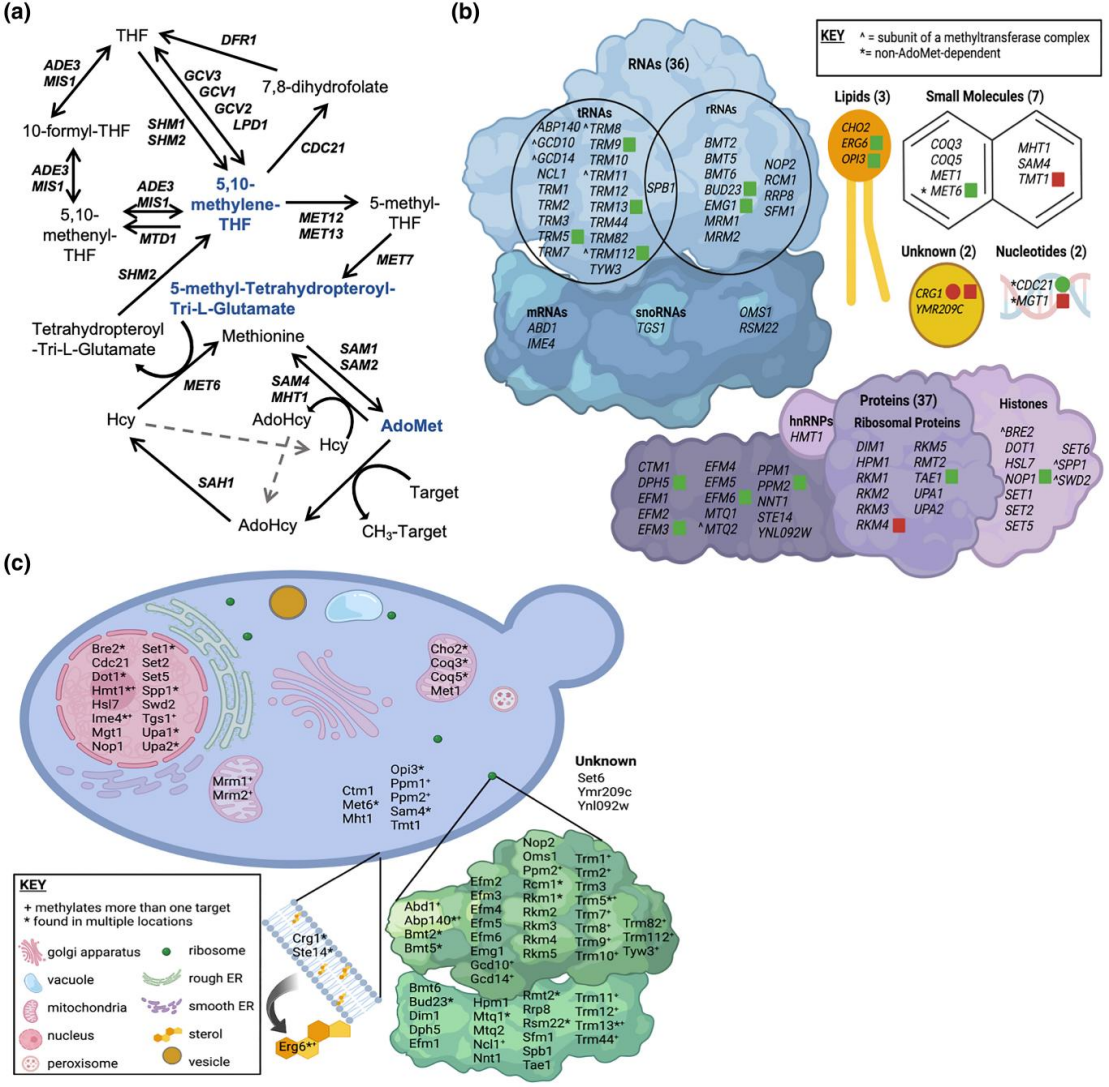
Methyl donors, methyltransferases, and methylation target molecules of *S. cerevisiae*. a) The methyl cycle in *S. cerevisiae* which produces the main methyl donor AdoMet, and portions of the folate cycle that produce the less frequently used methyl donors, 5,10-methylenetetrahydrofolate and 5-methyltetrahydrofolate. b) There are 87 identified methyltransferases in budding yeast, with the target macromolecule identified for most. The main targets are proteins and RNAs, followed by small molecules, lipids, and nucleotides, with 2 methyltransferases with unconfirmed targets. c) The methyltransferases are found in a wide variety of locations in the cell. The “*” next to methyltransferase names identify enzymes that are active in more than 1 location/organelle. The “+” identifies enzymes that methylate multiple target molecules. b and c) Created with BioRender.com.

*SAM1* and *SAM2* are paralogous genes in *Saccharomyces cerevisiae*, which encode proteins responsible for the synthesis of S-Adenosylmethionine (AdoMet), the major methyl donor in all cells (Fig. 1a). Cells with either *sam1* or *sam2* deletions are able to survive, however, the double homozygous deletion of *sam1* and *sam2* renders cells inviable (Thomas and Surdin-Kerjan 1997). *SAM* gene mutations have been identified to alter AdoMet abundance; *sam1*-deficient cells have been found to have increased AdoMet levels, whereas *sam2*-deficient cells have decreased AdoMet levels (Hoffert *et al*. 2019). Methylation events regulate many cellular processes, and in *S. cerevisiae*, the cellular targets regulated include RNAs, proteins, lipids, nucleotides, and small molecules (Fig. 1b) (Cheng and Roberts 2001).

There are 87 identified methyltransferases in *S. cerevisiae*, all but 3 are AdoMet-dependent, and 84 contain known or putative substrates (Fig. 1b). Of the AdoMet-dependent methyltransferases, 42.9% target RNA molecules, 44% target proteins, 3.6% have lipid targets, 7.1% target small molecules, and 2.4% remain with their target unidentified (Petrossian and Clarke 2009; Roguev *et al*. 2001; Webb *et al*. 2010; Carman and Han 2011; Wlodarski *et al*. 2011; Soares and Buratowski 2012; Sharma *et al*. 2014; Lv *et al*. 2015; Al-Hadid, White, and Clarke 2016; Jethmalani and Green 2020; Ismail *et al*. 2022; Chou *et al*. 2023). Methyltransferases play roles in a wide variety of processes including translation and ribosome synthesis, RNA processing, DNA maintenance, amino acid metabolism, respiration, vesicle transport, autophagy, lipid homeostasis, sterol synthesis, and sulfur and nitrogen reduction (Fig. 1c) (Fauchon *et al*. 2002; Webb *et al*. 2010; Wlodarski *et al*. 2011; Alam *et al*. 2021; González-Rodríguez *et al*. 2021). The wide range of targets, along with the large variety of impacts of the methylation events themselves, lead to broad effects in the cell and are not limited to impacting only a few pathways or cellular outcomes.

Beyond its use as a methyl donor, AdoMet plays a key role in other cellular and metabolic pathways, including glutathione (GSH), polyamine, and dNTP synthesis. AdoMet is produced from methionine (Met) and ATP. When an AdoMet-dependent methyltransferase uses AdoMet as a methyl donor and transfers off the methyl group, the byproduct is S-Adenosylhomocysteine (AdoHcy) (Fig. 1a). AdoHcy reacts to form homocysteine (Hcy) which can then be converted back to methionine using a methyl donor from the folate cycle, or instead be used in the transsulfuration pathway, responsible for production of GSH, cysteine, and important signaling molecules. Glutathione has a key role in cellular defense against reactive oxygen species (ROS) (Sbodio, Snyder, and Paul 2019). Homocysteine conversion back to Met requires tetrahydrofolate (THF) vitamers from the folate cycle (Suliman *et al*. 2005). The folate cycle is further important in synthesis of amino acids, nucleic acids, and nitrogenous bases; for instance, through supplying the methyl donor for conversion of dUMP to dTMP (Tjong, Dimri, and Mohiuddin 2022). Altered dNTP levels have been shown to cause a variety of problems that results in increased damage to DNA and chromosomal instability (Pai and Kearsey 2017). The aminopropylation pathway metabolizes AdoMet to synthesize polyamines, which include putrescine, spermidine, and spermine, necessary for cell growth (Giulidori *et al*. 1984). Spermidine and spermine are crucial for stabilizing DNA, RNA, and the cellular membrane, as well as having roles in signal transduction, stress response and homeostasis, RNA synthesis, and protein interactions (Miller-Fleming *et al*. 2015).

Our group has previously found that AdoMet concentration perturbances, due to the independent loss of *sam1* or *sam2*, result in changes to the stability of the genome as measured with chromosome stability assays (Hoffert *et al*. 2019). Interestingly, *sam1* deletion results in increased AdoMet concentrations and a more stable genome while the removal of *sam2* decreased AdoMet concentrations and resulted in a less stable genome. Thus, despite encoding the same enzyme, with 92% protein sequence identity, alternate functionality and/or regulation mechanisms must apply to each gene/protein independently. Further, in *sam2Δ*/sam2*Δ* cells, we have observed an additional downregulation of *SAM1*, therefore these cells represent both reduced AdoMet concentrations and would have reduced Sam1- and Sam2-dependent processes (Hoffert *et al*. 2019). Whereas, the *sam1Δ*/sam1*Δ* cells have abundant AdoMet and would only be reduced in Sam1-dependent processes. Due to the wide array of pathways in the cell that are impacted by AdoMet availability, including methylation events and molecules produced from products of the methyl cycle, we sought to broadly characterize the multitude of changes occurring in cells due to *sam1* and *sam2* gene deletions. To accomplish this, we have undertaken phenotypic profiling and gene expression analysis in these mutant strains. Phenotypic profiling involved the Phenotypic Microarray system from BiOLOG, and characterizing growth differences of mutant and wildtype strains in over 1,000 different conditions (Bochner 2003). Observed growth differences were then explored to understand the mechanism of action of the causative condition and its relation to methylation events, methyl cycle products, and/or AdoMet availability. We also undertook RNA-Sequencing (RNA-seq) experiments to understand the impacts of AdoMet availability on gene expression. Many methylation events impact gene expression, including via histone methylation and mRNA methylation. Therefore, exploration of differential gene expression in our mutant strains also allows us to characterize impacted genes/pathways with no intrinsic relationship to methylation, the methyl cycle, or AdoMet, beyond their regulation. Further, we sought to link these 2 methodologies, aiming to align these data sets to elucidate where gene expression differences explain altered phenotypes. Through the combination of these methodologies, we have built a more comprehensive understanding of the changes occurring due to the *sam1* and *sam2* deletions and are able to hypothesize which of these altered pathways might be contributing to the observed impacts on genome stability.

## Materials and methods

### Strains

The *S. cerevisiae* strains used in this study are based on our W303 wildtype strain: MAT a/α, *leu2-3/leu2-3*, *his3-Δ200/his3-Δ200*, *trp1-Δ1/trp1-Δ1*, *lys2-801/LYS2*, *ura3-52/ura3-52*, *can1-100/CAN1*, *ade2-101/ade2-101*, *2x [CF:(ura3::TRP1*, *SUP11*, *CEN4*, *D8B)]*. The homozygous deletant strains for *SAM1* and *SAM2* were created as described in Hoffert *et al*. (2019). Briefly, *SAM1* was knocked out with a KANMX cassette conferring resistance to G418: *sam1*::KANMX, and *SAM2* was knocked out with a *LEU2* cassette correcting the leucine auxotrophy: *sam2*::*LEU2*. Throughout this manuscript, these strains are referenced by their *SAM1* and *SAM2* gene status: wildtype, *sam1Δ*/sam1*Δ*, and *sam2Δ*/sam2*Δ*.

### RNA extraction and quality assessment

To prepare our yeast strains for RNA extraction, samples were grown in synthetic complete (SC) media altered to match their genotypic requirements (*sam1Δ*/sam1*Δ*: addition of G418, and *sam2Δ*/sam2*Δ*: removal of leucine). Samples were grown at 30°C overnight with agitation to mid-exponential phase (OD600 = 0.5–0.6). RNA extraction then follows the RNA Isolation procedure from the TRIzol Reagent protocol (Ambion Cat #15596-018) with minor modifications for cell disruption. Briefly, 1.5 mL of each culture was spun down at 14,000 rpm for 1 min. The media was decanted and 750 µL of TRIzol was added. Samples were resuspended via pipetting and 250 µL of SIGMA acid-washed glass beads, 425–600 µm in size, were added. Samples were bead-beaten for 15 s, then rested on ice for 5 min, beaten again for 25 s, rested on ice for 5 min, then beaten for a final 15 s and placed back on ice. The RNA precipitation, RNA wash, and RNA resuspension steps of the TRIzol protocol were then followed. After pellets were dissolved in RNAase-free ddH_2_O and incubated at 60°C for 15 min, they were stored at −80°C until purified. RNA Purification was completed using the RNeasy RNA Cleanup protocol and spin columns, estimating for up to 1 mg of RNA (Midi kit), from Qiagen, with DNase treatment on the columns and removal by subsequent washes. RNA quantity and quality were assessed via Nanospec following the manufacturer’s guidelines. RNA quality was further assessed by running 1 µg of each sample on a 1% TAE gel with bleach at 100 V for 40 min (Aranda, LaJoie, and Jorcyk 2012).

### RNA-Sequencing library preparation

Samples were sent to the University of Louisville Genomics Core Facility for RNA-seq. The TruSeq Stranded mRNA LT Sample Prep Kit—Set A and Set B with poly-A enrichment was used to prepare a library for RNA-seq. Briefly, polyadenylated RNA was purified, fragmented, and primed. First and second cDNA strands were synthesized and purified, and 3′ ends were adenylated. Then, the samples were barcoded with Illumina TruSeq adapters and the dsDNA fragments were enriched via PCR. The samples were purified, and quality was assessed using the Agilent DNA 1000 kit. The fragment size of the samples was about 300–350 bp in length. Sample quantity was assessed via a standard curve method from qPCR using DNA standards from the KAPA Library Quantitation Kit for Illumina Platforms. Next, the samples were normalized and pooled. Finally, the libraries were denatured and diluted in preparation for sequencing.

### RNA-Sequencing

An Illumina NextSeq 500, using NextSeq 500/550 1 × 75 cycle High Output Kit v2, was used to sequence the prepared library. Triplicates of 3 strains were analyzed: wildtype, *sam1Δ*/sam1*Δ*, and *sam2Δ*/sam2*Δ*. Thirty-six single-end raw sequencing files (.fastq) were analyzed using the tuxedo suite pipeline. The raw data were condensed into 1 single-end .fastq file for each triplicate giving 9 files representing each mutant strain (Cock *et al*. 2010). Quality control (QC) was performed using FastQC (version 0.10.1) and it was determined that trimming the samples was unnecessary (Andrews 2014). Tophat2 (version 2.0.13) was used to align sequences to the *S. cerevisiae* reference genome assembly (Saccharomyces_cerevisiae.R64-1-1.dna.toplevel.fa) (Kim *et al*. 2013). The tuxedo suite of programs, including cufflinks-cuffdiff2 (version 2.2.1), was used to identify differentially expressed genes (DEGs) between each mutant strain and wildtype (Trapnell *et al*. 2013; 2012). Cutoff limits for significance were as follows: *P* ≤ 0.05, −0.58 ≤ log2 Fold Change (log2FC) ≥ 0.58. A multiple comparison correction (MCC) was also applied, and *q*-value determined. These data can be found in Supplementary Table 1. As we are interested in investigating pathways that might involve multiple DEGs, we are including discussion of all gene expression differences that meet the *P* ≤ 0.05, −0.58 ≤ log2FC ≥ 0.58 cutoffs, rather than the more stringent MCC *q*-values. In this manner, we can visualize pathways where multiple *P*-value level significant DEGs are found, lending support to these not being false positive results. Global representation of the RNA-seq findings can be visualized in the Principal Component Analysis (PCA) and Heatmap of the DEGs from each strain in Supplementary Fig. 1. In the PCA, DEGs from the RNA-seq experiment comparisons between wildtype vs *sam1Δ*/sam1*Δ* and vs *sam2Δ/sam2Δ* strains were utilized. The raw read counts were transmuted using the Variant-Stabilizing Transformation (VST) function provided by the R library DeSeq2 (Love, Huber, and Anders 2014). The PCA was calculated using DeSeq2's plotPCA function and then plotted using ggplot2. A Heatmap was generated by once again running the raw read counts through VST and then centering and scaling them using the base R function “scale”. One gene, YNR075C-A, had a missing value for one of the samples and was removed. The plotting of this data was achieved using the Heatmap function from the R library ComplexHeatmap (Gu, Eils, and Schlesner 2016; Gu 2022).

### Phenotypic Microarray

Phenotypic Microarray (PM) plates were obtained from BiOLOG to test our mutant strains for phenotypic differences under a variety of conditions. Each well contains the necessary requirements for growth as well as different substrates or differing concentrations of the same substrate, testing a variety of conditions from metabolism to drug sensitivity. Substrate contents of each well are listed in Supplementary Table 5; concentrations are proprietary and confidential to BiOLOG. PM1 and PM2A MicroPlates test carbon sources, PM3B MicroPlate tests nitrogen sources, PM4A MicroPlate tests phosphorous and sulfur sources, PM5 MicroPlate tests nutrient supplements, PM6-8 MicroPlates test peptide nitrogen sources, PM9 MicroPlate tests osmolytes, PM10 tests pH, and PM21D, PM22D, PM23A, PM24C, and PM25D test for chemical sensitivities. All tests were conducted in duplicate for all 3 strains of interest.

Plates were inoculated per manufacturer instructions and the genotypes of our strains. Briefly, a stock solution for PM1-8 was prepared by adding 5 mL of His (2.4 mg/mL), Trp (4.8 mg/mL), Ura (2.4 mg/mL), and Leu (7.2 mg/mL) to 580 mL of ddH_2_O. A stock solution for PM9+ was prepared by adding 5 mL of His (2.4 mg/mL), Trp (4.8 mg/mL), Ura (2.4 mg/mL), and Leu (7.2 mg/mL) to 580-mL SC-5 solution. The inoculating fluid for PM1-2 was prepared by adding 20 mL of IFY-0 (1.2×), 0.32 mL of dye mix D (75×), and 3.18 mL of ddH_2_O. The inoculating fluid for PM3-8 was prepared by adding 60 mL of IFY-0 (1.2×), 0.96 mL of dye mix D (75×), 3 mL of D-glucose (24×), and 6.54 mL of ddH_2_O. The inoculating fluid for PM9+ was prepared by adding 70 mL of SC medium (1.2×), 0.84 mL of dye mix E (100×), and 7.91 mL of ddH_2_O.

Each yeast strain was streaked on separate BUY agar plates from BiOLOG and grown for 24 h at 30°C then subcultured to a new BUY plate for an additional 24 h. Cells from subculture plates were transferred into the previously prepared stock solutions to obtain uniform suspensions with a turbidity of 62% transmittance. A total of 0.50 mL of the cell suspension was added to 23.5 mL of PM1,2 inoculating fluid, and 100 µL was added to each well of PM1 and PM2. A total of 1.50 mL of the cell suspension was added to 70.50 mL of PM3-8 inoculating fluid, and 100 µL was added to each well of PM3-8. A total of 1.75 mL of the cell suspension was added to 82.25 mL of PM9+ inoculating fluid, and 100 µL was added to each well of PM9-10, 21-25. A BioTek ELx800 microplate reader was used to obtain optical densities (OD) of the wells in each plate at 0 h, 24, 48, and 72 h. All plates were sealed with parafilm and incubated at 30°C with agitation between OD readings. Prior to each OD reading, the cells were resuspended via pipetting. OD readings for biological replicates at each timepoint were plotted to generate growth curves. Any growth curves where either the mutant strain or wildtype strain biological replicates were not consistent were deemed inconclusive. Significant PM growth curves that were used in this manuscript were depicted using GraphPad Prism version 9.5.1 for Windows, GraphPad Software. Graphs were generated using the third order polynomial (cubic) equation using the Gompertz-growth curve model, and each graph was interpolated with a 95% confidence interval. Goodness-of-fit was measured using nonlinear regression analysis with GraphPad Prism. Additionally, 1 condition from each story (A–F) was selected for verification of the growth difference phenotype. Growth was tested over a 72-h period, grown at 30°C, with absorbance readings taken ever 17 and 38 s. Inoculations began with ∼200 cells of each strain of interest into YPD supplemented with the drug/condition to be tested at the following concentrations, fluconazole 0.48 µg/mL, cisplatin 1.7 µg/mL, sodium selenite 198 µg/mL, L-glutamic acid γ-monohydroxamate 597 µg/mL, 6-azauracil 133 µg/mL, and tamoxifen 0.014 µg/mL. Growth curves were generated as above (Supplementary Fig. 2). All absorbance data collected and growth curves generated for this study can be found in Supplementary Tables 6 and 7.

### Pathway analysis

To determine impacted pathways by conditions in the PM wells of interest, and find and map all involved genes, we executed a series of steps. First, we identified the specific targets of the conditions where growth differences of interest were observed. The specific effects of each condition or drug were carefully researched through databases, including PubMed, DrugBank, and the Saccharomyces Genome Database (Issel-Tarver *et al*. 2002; Wishart *et al*. 2006; 2018; Cherry *et al*. 2012). We extensively examined each condition and their associated mechanism of action through exhaustive literature searches focusing both on *S. cerevisiae* specific impacts and those in other systems. Next, we moved to researching what genes might be involved, again using literature searches in PubMed as well as the articles already identified. If the mechanism of action research yielded specific enzymes or pathways that were impacted, these were then researched using the Saccharomyces Genome Database, and cited literature within was reviewed. To fully map pathways around identified genes or processes, the Saccharomyces Genome Database YeastPathways biological maps and BRENDA Enzyme Database pathway maps were utilized (Schomburg *et al*. 2000; Issel-Tarver *et al*. 2002; Cherry *et al*. 2012; Chang *et al*. 2021). Our own representation of the pathway was then created that included all involved genes from all sources.

## Results

### RNA-Sequencing

RNA-seq revealed 134 DEGs in the *sam1Δ*/sam1*Δ* mutant strain. Specifically, there were 59 upregulated genes and 75 downregulated genes at cutoff values of *P* ≤ 0.05, −0.58 ≤ log2FC ≥ 0.58, (Supplementary Table 1). Gene ontology analysis of these DEGs revealed enrichment for several terms across the 3 domains (Table 1). When looked at altogether, it appears that the deletion of both copies of *sam1* leads to differential expression of genes that play roles in protein and xenobiotic metabolism as well as transmembrane transport of ions.

**Table 1.** GOConsortium gene ontology categorization of differentially expressed genes from RNA-Sequencing.

Strain	GO category	GO: ID	Description	±	*P*-value	FDR
*sam1Δ/sam1Δ*	Biological process	GO:0006805	Xenobiotic metabolic process	+	1.23e^−04^	3.56e^−02^
		GO:0044718	Siderophore transmembrane transport	+	1.83e^−04^	4.76e^−02^
		GO:0009063	Cellular amino acid catabolic process	+	1.34e^−04^	3.67e^−02^
		GO:0044267	Cellular protein metabolic process	+	8.91e^−05^	2.73e^−02^
		GO:0010467	Gene expression	+	7.39e^−06^	9.63e^−03^
	Molecular function	GO:0015293	Symporter activity	+	4.73e^−05^	3.00e^−02^
		GO:0015075	Ion transmembrane transporter activity	+	4.46e^−05^	3.77e^−02^
	Cellular component	GO:0000786	Nucleosome	+	4.65e^−04^	4.95e^−02^
		GO:0005887	Integral component of plasma membrane	+	4.34e^−06^	4.61e^−03^
		GO:0000324	Fungal-type vacuole	+	4.51e^−05^	1.60e^−02^
*sam2Δ/sam2Δ*	Biological process	GO:0006526	Arginine biosynthetic process	+	2.89e^−05^	5.57e^−03^
		GO:0006591	Ornithine biosynthetic process	+	8.69e^−05^	1.37e^−02^
		GO:0000097	Sulfur amino acid biosynthetic process	+	1.09e^−04^	1.58e^−02^
		GO:0072528	Pyrimidine-containing compound biosynthetic process	+	4.00e^−04^	4.97e^−02^
		GO:0006555	Methionine metabolic process	+	2.63e^−04^	3.43e^−02^
		GO:0046942	Carboxylic acid transport	+	2.14e^−05^	4.64e^−03^
		GO:0019693	Ribose phosphate metabolic process	+	2.79e^−04^	3.54e^−02^
		GO:0006753	Nucleoside phosphate metabolic process	+	7.95e^−05^	1.34e^−02^
		GO:0042254	Ribosome biogenesis	+	9.32e^−05^	1.43e^−02^
		GO:0006897	Endocytosis	−	6.73e^−06^	1.95e^−03^
		GO:0032197	Transposition, RNA-mediated	−	1.25e^−04^	1.72e^−02^
	Molecular function	GO:0046943	Carboxylic acid transmembrane transporter activity	+	2.09e^−05^	2.66e^−02^
		GO:0008514	Organic anion transmembrane transporter activity	+	5.63e^−05^	2.66e^−02^
		GO:0043167	Ion binding	+	9.12e^−05^	4.63e^−02^
		GO:0003824	Catalytic activity	+	3.07e^−06^	7.79e^−03^
	Cellular component	GO:0110165	Cellular anatomical entity	+	1.03e^−05^	1.09e^−02^

GOConsortium was used to analyze *sam1Δ/sam1Δ* and *sam2Δ/sam2Δ* DEGs for enriched and underrepresented biological process, molecular function, and cellular component gene ontology terms at a *P*-value and FDR cutoff of 0.05 (Ashburner *et al*. 2000; Mi *et al*. 2019; The Gene Ontology Consortium *et al*. 2023). Overrepresented terms are indicated with “+” whereas underrepresented terms are indicated with “−”. The most specific subclass of enriched terms for each category is shown. A complete list of enriched terms, including parent classes, can be found in Supplementary Table 2.

The *sam2Δ*/sam2*Δ* mutant strain had 876 DEGs, consisting of 405 upregulated genes and 471 downregulated genes (*P* ≤ 0.05, −0.58 ≤ log2FC ≥ 0.58) (Supplementary Table 1). Gene ontology analysis of all DEGs from *sam2Δ*/sam2*Δ* cells revealed several enriched terms across the 3 domains (Table 1), including the biological process of “amino acid and deoxynucleoside triphosphate (dNTP) metabolism”, and molecular functions including “transmembrane transport”, “ion binding”, “protein and xenobiotic metabolism”, and “catalytic activity”. Three AdoMet-dependent methyltransferases were upregulated: *RKM4*, *CRG1*, and *TMT1*, while 15 AdoMet-dependent methyltransferases were significantly downregulated: *ERG6*, *OPI3*, *TAE1*, *EFM3*, *EFM6*, *DPH5*, *PPM2*, *NOP1*, *BUD23*, *NOP2*, *EMG1*, *TRM5*, *TRM9*, *TRM13*, and *TRM112* (Fig. 1b) [*P* ≤ 0.05, −0.58 ≤ log2FC ≥ 0.58, (Supplementary Table 1)]. This is of interest because the vast majority of AdoMet-dependent methyltransferases are known to be regulated by transcription factors and/or chromatin modifications (Mendiratta *et al*. 2006; Ellahi, Thurtle, and Rine 2015; Monaco *et al*. 2018; Kishkevich *et al*. 2019).

In comparing the DEGs from each homozygous knockout strain, 59 genes (6.2%) were found in both. Forty-four of these 59 genes showed the same expression pattern in each mutant strain (26 with decreased expression, 18 with increased expression), whereas 15 genes showed opposite expression patterns between the 2 mutant strains (11 *sam1Δ*/sam1*Δ* decreased but *sam2Δ*/sam2*Δ* increased, and 4 *sam1Δ*/sam1*Δ* increased but *sam2Δ*/sam2*Δ* decreased). A list of these genes can be found in Supplementary Table 3.

### Phenotypic Microarray

We utilized 15 PM plates to characterize phenotypic growth differences in our mutant strains (PM1-10, PM21D, PM22D, PM23A, PM24C, and PM25D) (Bochner 2003). There were 1,440 total wells with 13 serving as either positive or negative controls. Many wells contained the same substrate but at varying concentrations. With those instances counting as a single condition, and controls removed, our wildtype and mutant strains were exposed to a total of 1,005 completely different test conditions during growth. When analyzing growth to then define different phenotypes, 3 parts of the growth curves were assessed as was done in Fernandez-Ricaud *et al*. (2007). These parts include the adaptation time (time to start of growth), rate of growth, and efficiency of growth (time to saturation and maximum OD).

The *sam1Δ*/sam1*Δ* mutant strain, which exhibits increased AdoMet levels, showed patterns of growth that differed from the wildtype strain in 146 total wells that encompassed 98 different conditions tested, representing altered growth in 9.75% of the conditions (Supplementary Table 4). The largest grouping (94.9%) impacted rate of growth, whereas 59.2% altered efficiency, and 45.1% impacted adaptation time (Table 2). The *sam2Δ*/sam2*Δ* mutant strain showed altered growth in 206 total wells that encompassed 148 different conditions (Supplementary Table 4). Thus, the full loss of *sam2* expression, which results in decreased AdoMet levels, has wider phenotypic impacts changing growth patterns in 14.7% of conditions tested. In the conditions that had differing growth, the largest group (95.9%) impacted growth rate, whereas 48.0% altered efficiency of growth and 39.2% impacted adaptation time (Table 2).

**Table 2.** Characterization of growth differences in each mutant strain using Phenotypic Microarray plates from BiOLOG.

Strain	Growth descriptor	Increased	Decreased	Total	Total conditions with altered growth
*sam1Δ/sam1Δ*	Adaptation time	41 (41.8%)	4 (4.1%)	45 (45.1%)	98
	Rate of growth	3 (3.1%)	89 (90.8%)	92 (94.9%)	
	Efficiency of growth	2 (2.0%)	56 (57.1%)	58 (59.2%)	
*sam2Δ/sam2Δ*	Adaptation time	6 (4.1%)	52 (35.1%)	58 (39.2%)	148
	Rate of growth	129 (87.2%)	13 (8.8%)	142 (95.9%)	
	Efficiency of growth	56 (37.8%)	15 (10.1%)	71 (48.0%)	

Growth patterns were analyzed for each condition and growth descriptors were applied where mutant strains showed differences. Graphs were not interpreted if biological replicate data for a given strain were not consistent. A single condition could be counted for more than 1 growth descriptor category.

Fifty-nine conditions were identified as impacting growth in both homozygous knockout mutant strains. Of these 59 conditions, 13 resulted in growth patterns of a similar nature (both more sensitive or more resistant) in both the *sam1Δ*/sam1*Δ* and *sam2Δ*/sam2*Δ* mutant strains compared to wildtype: D-galactose, D-xylose, D-fructose, a-D-glucose, L-lyxose, D-glucosamine, 5-keto-D-gluconic acid, EDTA, sodium selenite, chromium chloride, blasticidin S, potassium chromate, and B-chloro-L-alanine. We hypothesize that the pathways impacted by these conditions are not related to the increased and decreased AdoMet levels or increased and decreased chromosome stability seen in the *sam1Δ*/sam1*Δ* and *sam2Δ*/sam2*Δ* strains, respectively, since the growth patterns are similar despite the different mutations. The remaining 46 conditions elicited growth patterns that were opposite in nature (i.e. one strain was more resistant while the other was more sensitive) between the *sam1Δ*/sam1*Δ* and *sam2Δ*/sam2*Δ* mutant strains. The mutant strains showed opposite altered growth patterns to varying concentrations of NaCl, ethylene glycol, 4% sodium formate, sodium lactate, sodium phosphate, pH 4.5 + L-tryptophan, pH 4.5 + hydroxyl-L-proline, pH 4.5 + D,L-alpha-amino-n-butyric acid, dodecyl trimethyl ammonium bromide, protamine sulfate, magnesium chloride, diamide, L-gluatamic acid G-monohydroxamate, L-arginine hydroxamate, glycine hydroxamate, 3-amino-1,2,4-triazole, D,L-serine hydroxamate, bleomycin, ammonium sulfate, doxycycline, glycine hydrocholoride, hydroxylamine, chromium(III) chloride, cobalt(II) chloride, sodium metaborate, caprylic acid, sodium cyanate, sodium cyanide, sodium thiosulfate, chlortetracycline, sodium metasilicate, 6-azauracil, EGTA, sodium pyrophosphate, cisplatin, aluminum sulfate, fluconazole, tamoxifen, miconazole, tobramycin, tetrazolium violet, 4-nitroquinoline-N-oxide, succinic acid, fumaric acid, palladium(II) chloride, ibuprofen, and chloroquine. In general, in these conditions, loss of *sam1* resulted in decreased growth rates and lowered efficiencies, whereas loss of *sam2* resulted in increased growth rates sometimes accompanied with increased growth efficiencies. These conditions that impact both strains, but in nonidentical ways (as well as those conditions that impact one, but not the other mutant strain), are of interest, as the pathways that are impacted might underlie the previously observed chromosome stability changes which also have opposite patterns. The list of conditions eliciting opposite growth patterns therefore served as a starting point in selecting areas to study further (sections A–F below).

We also explored overrepresentations of conditions eliciting growth differences compared to their prevalence in the PM plates based on mechanism of action. We saw that 21.4% of the conditions where *sam1Δ*/sam1*Δ* mutant cells showed altered growth had a mechanism of action relating to toxic ions (2.9% of conditions in the PM wells tested), 8.1% related to protein synthesis (1% of PM wells tested), 7.1% related directly to membrane stability (1.6% of PM wells tested), 17.3% related to osmolarity/osmotic sensitivity (7.6% of PM wells tested), and 3% related to tRNA synthetases (0.5% of PM wells tested). In the majority of these conditions (all but 2 wells across all categories), the *sam1Δ*/sam1*Δ* mutant cells were more sensitive than wildtype, most frequently materializing as a decreased growth rate and/or increased adaptation time. The conditions that elicited altered growth in the *sam2Δ*/sam2*Δ* mutant cells could be broadly categorized as having mechanisms of action of which 10.8% related to toxic ions (2.9% of conditions in the PM wells tested), 4.1% related to protein synthesis (1% of PM wells tested), 6.8% related directly to membrane stability (1.6% of PM wells tested), 4.6% related to respiration (0.7% of PM wells), 26.4% related to osmolarity/osmotic sensitivity (7.6% of PM wells tested), and 2.7% related to tRNA synthetases (0.5% of PM wells tested). In all but 4 wells across all these categories, the *sam2Δ*/sam2*Δ* mutant cells were more resistant than wildtype, most frequently materializing as an increased growth rate and/or decreased adaptation time. These overrepresented categories then served as an additional selection mechanism in narrowing down conditions to study further.

### Linking phenotypic changes with underlying gene expression differences

The use of RNA-seq and the PM allowed broad characterization of differences in the *sam1Δ*/sam1*Δ* and *sam2Δ*/sam2*Δ* mutant strains. Additional work was needed to try to identify how these mutations, and the resultant alterations in AdoMet concentration, led to the differences that were observed. Therefore, we next sought to link the data collected from our RNA-seq experiments to the phenotypic differences from the PM data, to define pathway alterations that led to significant growth changes for our mutants. To do this, we identified the mechanisms of action of the conditions of a particular PM well and then mapped the affected pathways. Then, we overlayed our DEG data to see if the *sam1Δ*/sam1*Δ* or *sam2Δ*/sam2*Δ* deletions altered expression of genes in the pathways. Finally, we sought to determine if the changes in expression of impacted pathway genes, impacts to the methyl cycle, altered AdoMet-dependent methyltransferase expression, or AdoMet concentration differences/availability, could explain the observed growth differences of the strains.

#### A. Lack of *sam2* confers resistance to antifungal azole drugs, while *sam1*-deficient cells show increased sensitivity

**Fig. 2.**
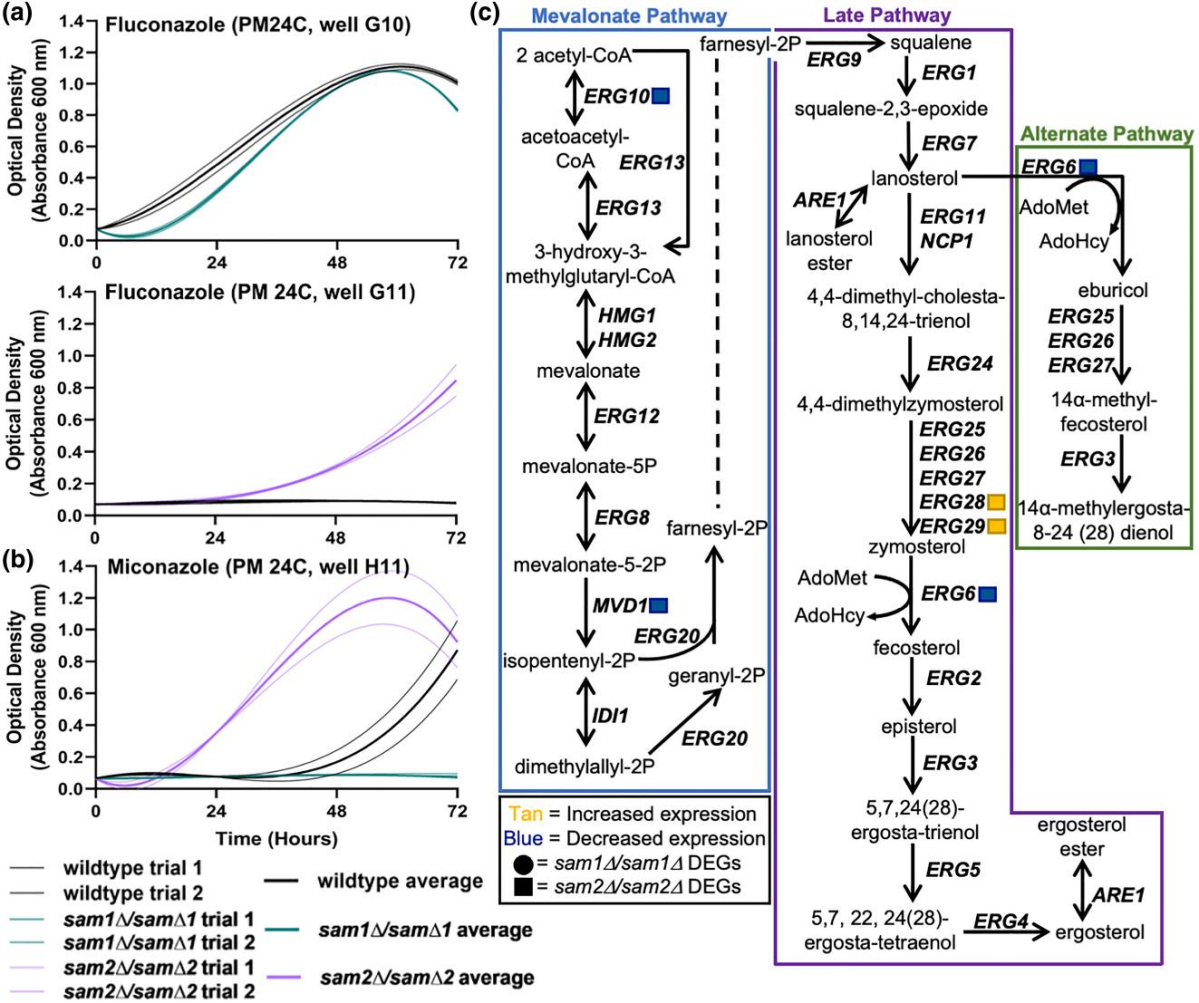
Growth curves of wildtype, *sam1Δ*/sam1*Δ*, and *sam2Δ*/sam2*Δ* cells in the presence of a) fluconazole and b) miconazole, and the impacted c) ergosterol biosynthesis pathway. Growth curves generated from timepoint data of the Phenotypic Microarray experiments. The independent trials for each strain are shown in a lighter weight line, while the average is shown in the same color in bold. a) Growth curves are shown for wildtype and *sam1Δ*/sam1*Δ* cells in the third highest concentration of Fluconazole (PM24C, G10) (R-squared Goodness-of-fit values: wildtype trial 1 = 0.9967, wildtype trial 2 = 0.9967, wildtype average = 0.9968, *sam1Δ*/sam1*Δ* trial 1 = 0.9737, *sam1Δ*/sam1*Δ* trial 2 = 0.9687, *sam1Δ*/sam1*Δ* average = 0.9713) and wildtype and *sam2Δ*/sam2*Δ* cells in the second highest concentration (PM24C, G11) (R-squared Goodness-of-fit values: wildtype no growth = NA, *sam2Δ*/sam2*Δ* trial 1 = 0.9980, *sam2Δ*/sam2*Δ* trial 2 = 0.9964, *sam2Δ*/sam2*Δ* average = 0.9977). b) Growth curves are shown for wildtype, *sam1Δ*/sam1*Δ*, and *sam2Δ*/sam2*Δ* cells in the second highest concentration of miconazole (PM24C, H11) (R-squared Goodness-of-fit values: wildtype trial 1 = 0.9956, wildtype trial 2 = 0.9743, wildtype average = 0.9894, *sam1Δ*/sam1*Δ* no growth = NA, *sam2Δ*/sam2*Δ* trial 1 = 0.9866, *sam2Δ*/sam2*Δ* trial 2 = 0.9526, *sam2Δ*/sam2*Δ* average = 0.9750). c) Production of ergosterol in *S. cerevisiae* begins with 2 acetyl-CoA molecules and produces additional sterols through the interconnected mevalonate, late, and alternate pathways. The output of the mevalonate pathway, farnesyl-2P, is the starting substrate for the late pathway. The gene names that encode the enzymes that function in compound conversion in the pathway are given by their standard names. *sam2Δ*/sam2*Δ* DEGs are represented by tan (increased expression) and blue (decreased expression) squares. RNA-Sequencing did not reveal any differentially expressed genes within this pathway between *sam1Δ*/sam1*Δ* and wildtype.

The 15 PM plates used contain 3 different azole fungicides (Supplementary Table 5), and we observed growth differences in our *sam1Δ*/sam1*Δ* and/or *sam2Δ*/sam2*Δ* mutant strains in all 3. Figure 2a and b shows representative growth curves of the wildtype, *sam1Δ*/sam1*Δ*, and *sam2Δ*/sam2*Δ* strains in response to these different azoles (for all growth curves, see Supplementary Tables 6 and 7). In fluconazole, the *sam1Δ*/sam1*Δ* mutant cells exhibited decreased growth rate, while the *sam2Δ*/sam2*Δ* mutant cells exhibited an increased growth efficiency and rate of growth (Fig. 2a). In miconazole, *sam1Δ*/sam1*Δ* mutant cells were inhibited from growth, while *sam2Δ*/sam2*Δ* mutant cells exhibited a decreased adaptation time and increased growth efficiency (Fig. 2b).

Cholesterol is the main sterol present in eukaryotic plasma membranes, except fungal plasma membranes, whose main sterol is ergosterol, but also use small amounts of zymosterol and other sterols (Zinser, Paltauf, and Daum 1993; Kodedová and Sychrová 2015). In *S. cerevisiae*, the ergosterol biosynthesis pathway has 3 sections: the mevalonate pathway, the late pathway, and the alternate pathway (Fig. 2c). Fluconazole, propiconazole, and miconazole are triazoles that selectively target lanosterol 14a-demethylase, which is a rate-limiting enzyme encoded by the gene *ERG11*. Both the late and alternate pathways involve an AdoMet-dependent methyltransferase encoded by the *ERG6* gene (Fig. 2c), responsible for the conversion of zymosterol into fecosterol and lanosterol into eburicol, respectively. Eburicol is ultimately converted to the toxic sterol, 14a-methylergosta-8,24(28)-dienol which inhibits proper membrane structure (Bhattacharya, Esquivel, and White 2018). *ERG10*, *MVD1*, *ERG6*, *ERG28*, and *ERG29* of the ergosterol synthesis pathway are DEGs in the *sam2Δ*/sam2*Δ* strain at cutoffs of *P* ≤ 0.05, −0.58 ≤ log2FC ≥ 0.58, (Supplementary Table 1); *ERG10*, *MVD1*, and *ERG6* are downregulated, while *ERG28* and *ERG29* are upregulated (Fig. 2c). Decreased *ERG6* expression in the *sam2Δ*/sam2*Δ* strain may lessen the input into the alternate pathway. Upregulation of *ERG28* and *ERG29* in the late pathway may increase zymosterol production, benefiting the cell as Erg6 functions directly downstream of these genes, and increasing its substrate when Erg6 is limited may increase conversion to fecosterol overall and facilitate completion of the late pathway to result in ergosterol production at more normal levels. Further, the significant reduction in AdoMet levels in *sam2Δ*/sam2*Δ* strain would limit methylation by Erg6 from reduced methyl donor availability. The decreased expression of *ERG6*, and the reduced concentration of AdoMet, can explain the resistance to azoles, as while Erg6 functions in both the late and alternate pathways it's reduced activity in the alternate pathway results in less 14a-methylergosta-8,24(28)-dienol production, which does not then build up to toxic levels. No ergosterol pathway genes emerge as DEGs in *sam1Δ*/sam1*Δ* mutants. However, this strain has increased AdoMet, and abundance of this methyl donor may influence the increased sensitivity to azoles, because while the *ERG6* gene is not increased, it's rate of activity may be enhanced by the increased AdoMet. Therefore, when Erg11 is targeted by fluconazole or miconazole, and cells shift to the alternate pathway in an attempt to survive, *sam1*-deficient cells may produce the toxic dienol faster than wildtype cells.

#### B. Lack of *sam2* confers resistance to cisplatin

**Fig. 3.**
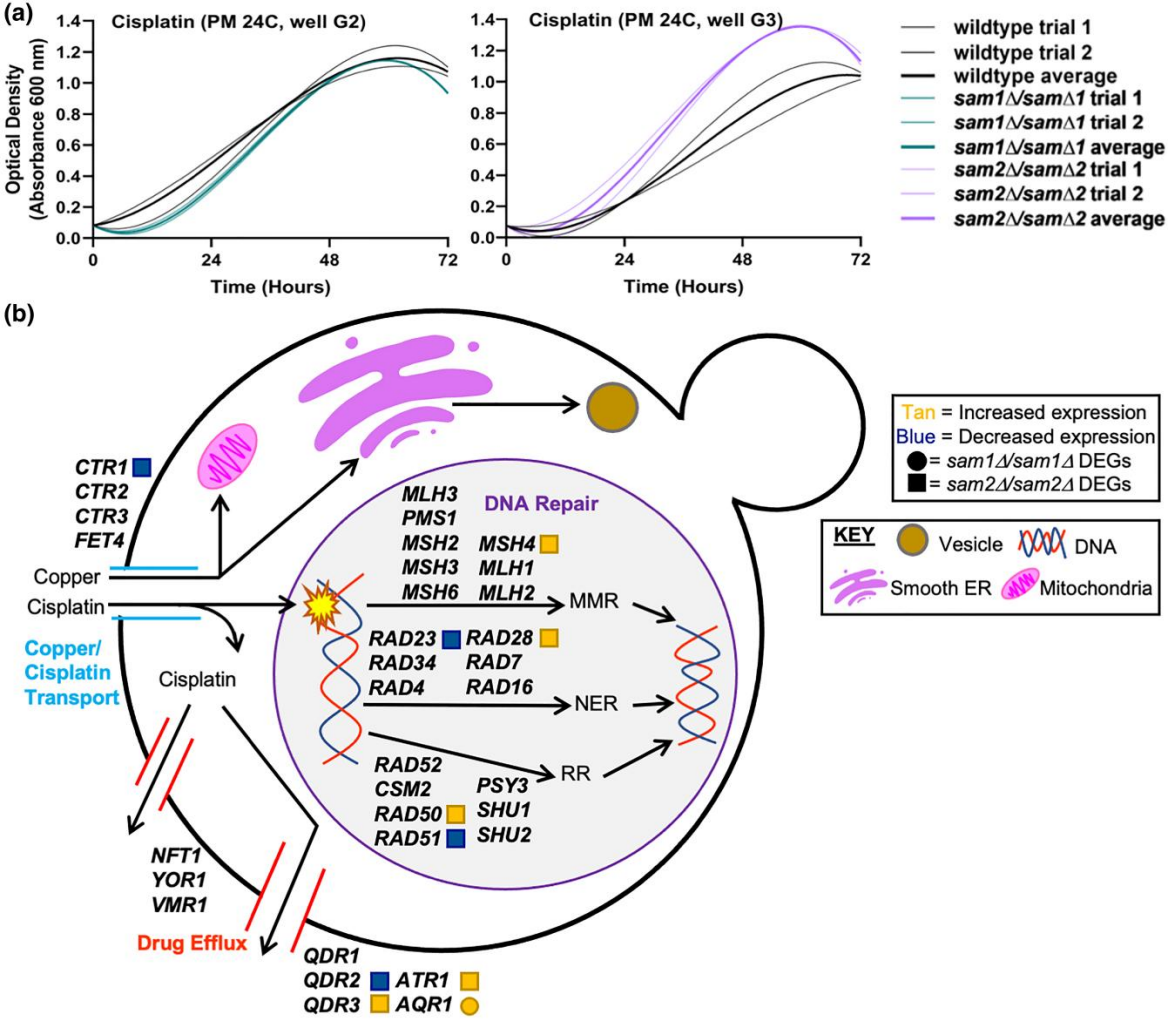
a) Growth curves of wildtype, *sam1Δ*/sam1*Δ*, and *sam2Δ*/sam2*Δ* cells in the presence of cisplatin, and the related b) copper/cisplatin transport pathway, DNA repair pathways, and drug efflux pathways. a) Growth curves generated from timepoint data of the Phenotypic Microarray experiments. The independent trials for each strain are shown in a lighter weight line, while the average is shown in the same color in bold. Growth curves are shown for wildtype and *sam1Δ*/sam1*Δ* cells in the third highest concentration of cisplatin (PM24C, G2) (R-squared Goodness-of-fit values: wildtype trial 1 = 0.9993, wildtype trial 2 = 0.9939, wildtype average = 0.9979, *sam1Δ*/sam1*Δ* trial 1 = 0.9844, *sam1Δ*/sam1*Δ* trial 2 = 0.9835, *sam1Δ*/sam1*Δ* average = 0.9840) and wildtype and *sam2Δ*/sam2*Δ* cells in the second highest concentration (PM24C, G3) (R-squared Goodness-of-fit values: wildtype trial 1 = 0.9950, wildtype trial 2 = 0.9994, wildtype average = 0.9971, *sam2Δ*/sam2*Δ* trial 1 = 0.9994, *sam2Δ*/sam2*Δ* trial 2 = 0.9903, *sam2Δ*/sam2*Δ* average = 0.9914). b) Uptake of copper and cisplatin in *S. cerevisiae* begins with import by membrane-associated transport proteins. Copper aids in several cellular processes in specialized organelles, such as the mitochondria, smooth endoplasmic reticulum, and vesicles. Cisplatin causes DNA damage that can be subsequently repaired by DNA repair pathways: MMR, NER, or RR. Cisplatin can be exported out of the cell by specialized drug efflux pumps. The gene names that encode the proteins that function at particular points in the pathway are given by their standard names. *sam2Δ*/sam2*Δ* DEGs are represented by tan (increased expression) and blue (decreased expression) squares. *sam1Δ*/sam1*Δ* DEGs are represented by tan (increased expression) and blue (decreased expression) circles.

Cisplatin, a known chemotherapeutic, is found in PM24C, wells G1-4, and we observed growth differences in both of our mutant strains in this condition. Figure 3a shows the growth curves of the wildtype, *sam1Δ/sam1Δ*, and *sam2Δ*/sam2*Δ* strains in response to cisplatin. Multiple concentrations were tested in different PM wells and data from a representative well are shown for each mutant. The *sam1Δ/sam1Δ* mutant cells show an increased adaptation time and lowered rate of growth, while the *sam2Δ/sam2Δ* mutant cells show an increased growth rate and efficiency, in comparison to wildtype, on average (Fig. 3a) (for all growth curves, see Supplementary Tables 6 and 7).

Cisplatin is a platinum-based drug, with genotoxic, cytostatic, and cytotoxic effects (Hannan, Zimmer, and Hazle 1984). We envisioned that cell sensitivity or resistance to cisplatin could be impacted through changes in the uptake of the drug, reaction/repair to damage caused by the drug, or altered export of the drug. DEGs from the *sam1Δ*/sam1*Δ* and *sam2Δ/sam2Δ* mutant strains include *CTR1* of the copper/cisplatin transport pathway, *MSH4*, *RAD50*, *RAD51*, *RAD28*, and *RAD23* of the DNA repair pathways, and *QDR2*, *QDR3*, *AQR1*, and *ATR1* of the drug efflux pathways, at cutoffs of *P* ≤ 0.05, −0.58 ≤ log2FC ≥ 0.58, (Supplementary Table 1) (Fig. 3b). *sam2Δ*/sam2*Δ* mutant cells exhibited decreased expression of *CTR1*. Previous studies have found that loss of *CTR1* results in almost complete resistance to cisplatin due to lowered ability of cisplatin transport into the cell (Abada and Howell 2011). We hypothesize that the lowered Ctr1 (at least partially) allows *sam2Δ/sam2Δ* cells to resist the deleterious effects of cisplatin due to decreased import and thus exposure of their DNA to the drug. Within the drug efflux pathway, 3 genes have been identified as responsible for exporting cisplatin only: *NFT1*, *YOR1*, and *VMR1*, each of which is part of the multidrug resistance-associated family of proteins (Fig. 3b) (Klein, Kuchler, and Valachovic 2011). Although none of these genes are DEGs, the *sam2Δ*/sam2*Δ* mutant cells do exhibit altered expression of a similar family of transporters that export several drugs including cisplatin: *QDR2*, *QDR3*, and *ATR1* (Fig. 3b). The Qdr1, Qdr2, Qdr3, and Atr1 proteins are multidrug/hydrogen ion antiporters, and are also known to be responsible for cisplatin resistance (Gömpel-Klein and Brendel 1990; Tenreiro *et al*. 2005). We hypothesize that because the *sam2Δ*/sam2*Δ* mutant cells exhibit upregulation of 2 of the multidrug export genes (*QDR3* and *ATR1*), they are likely able to pump out more cisplatin than wildtype or *sam1Δ*/sam1*Δ* mutant cells, which in combination with their decreased import underlies their increased growth rate and efficiency. Cisplatin causes damage to DNA by forming covalent bonds between the platinum ion and the N7 position of purine bases, which in turn forms a 1,2- or 1,3-intrastrand crosslink, rendering the DNA unable to be replicated or transcribed (Grossmann, Brown, and Moses 1999; Basu and Krishnamurthy 2010). The effects of cisplatin can be reversed through multiple DNA repair mechanisms, including nucleotide excision repair (NER), mismatch repair (MMR), and recombinational repair (RR) (He *et al*. 1996; Debrauwère *et al*. 2001; Goldfarb and Alani 2005; Rocha *et al*. 2018). Within these pathways, we observed several DEGs in the *sam2Δ/sam2Δ* mutant strain (Fig. 3b), including, NER: *RAD28* and *RAD23*, MMR: *MSH4*, and RR: *RAD50*, and *RAD51* (Fig. 3b). However, the mix of upregulation and downregulation of these genes makes it difficult to hypothesize an overall positive or negative impact; rather, we hypothesize that the growth changes we see in *sam2Δ/sam2Δ* cells are due to the changes in the import and efflux pathways. *QDR1* has a paralog, *AQR1*, which encodes a protein able to pump out cisplatin, and confer resistance to this drug (Kapetanakis *et al*. 2021). The *sam1Δ*/sam1*Δ* mutant cells contain upregulated *AQR1*. We found no additional DEGS in the *sam1Δ*/sam1*Δ* mutant strains in any of these pathways.

#### C. Lack of *sam2* leads to altered growth in conditions impacting glutathione and ROS

**Fig. 4.**
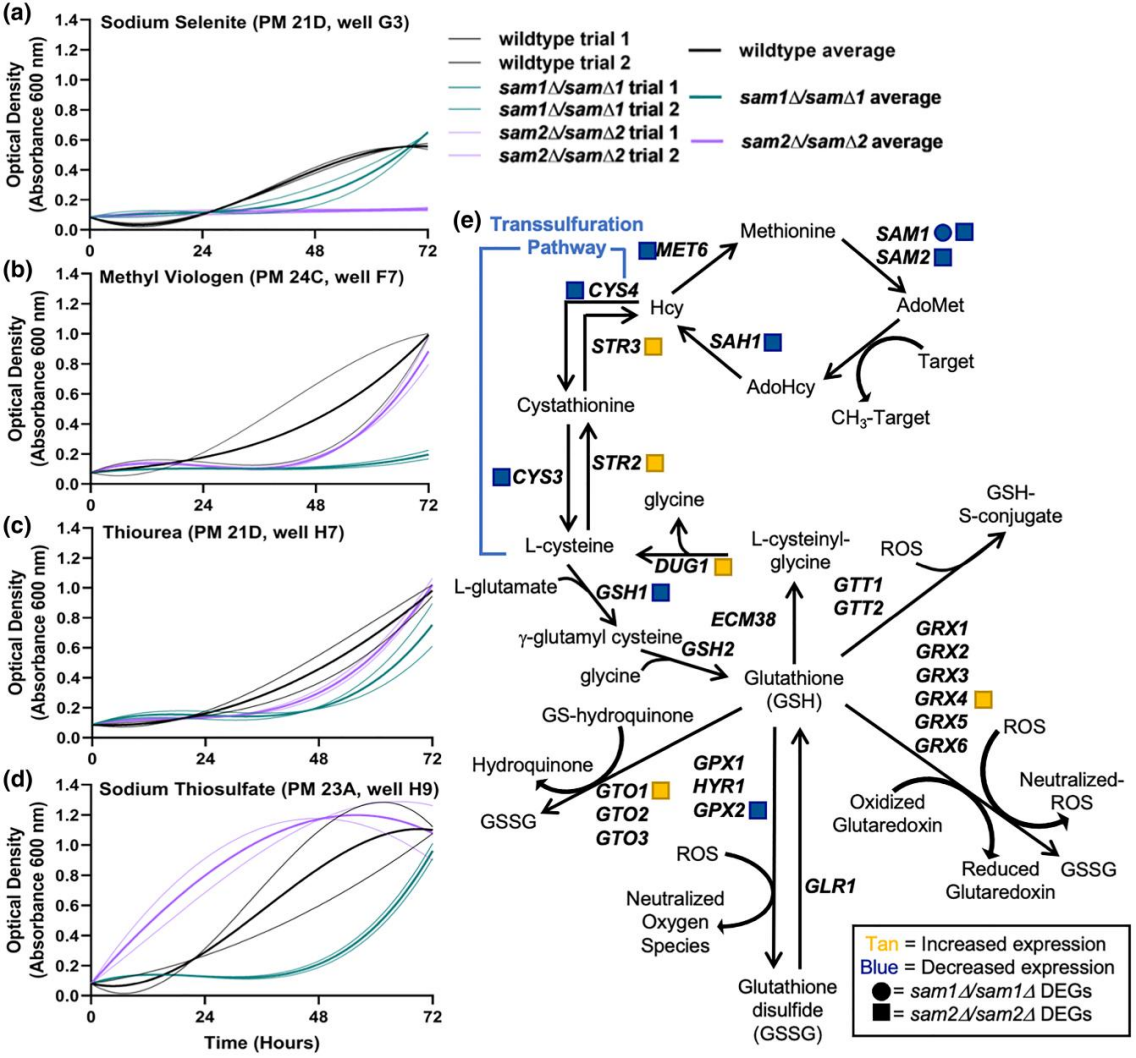
Growth curves of wildtype, *sam1Δ*/sam1*Δ*, and *sam2Δ*/sam2*Δ* cells in the presence of a) sodium selenite, b) methyl viologen, c) thiourea, and d) sodium thiosulfate, and the impacted e) glutathione biosynthesis pathway. Growth curves generated from timepoint data of the Phenotypic Microarray experiments. The independent trials for each strain are shown in a lighter weight line, while the average is shown in the same color in bold. Growth curves are shown for wildtype, *sam1Δ*/sam1*Δ*, and *sam2Δ*/sam2*Δ* cells in a) the second highest concentration of sodium selenite (PM21D, G3) (R-squared Goodness-of-fit values: wildtype trial 1 = 0.9803, wildtype trial 2 = 0.9657, wildtype average = 0.9728, *sam1Δ*/sam1*Δ* trial 1 = 0.9599, *sam1Δ*/sam1*Δ* trial 2 = 0.9955, *sam1Δ*/sam1*Δ* average = 0.9841, *sam2Δ*/sam2*Δ* no growth = NA), b) the second highest concentration of methyl viologen (PM24C, F7) (R-squared Goodness-of-fit values: wildtype trial 1 = 0.9982, wildtype trial 2 = 0.9762, wildtype average = 1.000, *sam1Δ*/sam1*Δ* trial 1 = 0.8583, *sam1Δ*/sam1*Δ* trial 2 = 0.9162, *sam1Δ*/sam1*Δ* average = 0.8940, *sam2Δ*/sam2*Δ* trial 1 = 0.9771, *sam2Δ*/sam2*Δ* trial 2 = 0.9793, *sam2Δ*/sam2*Δ* average = 0.9767), c) the second highest concentration of thiourea (PM21D, H7) (R-squared Goodness-of-fit values: wildtype trial 1 = 0.9977, wildtype trial 2 = 0.9982, wildtype average = 0.9986, *sam1Δ*/sam1*Δ* trial 1 = 0.9398, *sam1Δ*/sam1*Δ* trial 2 = 0.9793, *sam1Δ*/sam1*Δ* average = 0.9645, *sam2Δ*/sam2*Δ* trial 1 = 0.9948, *sam2Δ*/sam2*Δ* trial 2 = 0.9883, *sam2Δ*/sam2*Δ* average = 0.9923), and d) the second highest concentration of sodium thiosulfate (PM23A, H9) (R-squared Goodness-of-fit values: wildtype trial 1 = 0.9935, wildtype trial 2 = 0.9995, wildtype average = 0.9985, *sam1Δ*/sam1*Δ* trial 1 = 0.9843, *sam1Δ*/sam1*Δ* trial 2 = 0.9776, *sam1Δ*/sam1*Δ* average = 0.9812, *sam2Δ*/sam2*Δ* trial 1 = 0.9992, *sam2Δ*/sam2*Δ* trial 2 = 0.9414, *sam2Δ*/sam2*Δ* average = 0.9923). e) Production of glutathione in *S. cerevisiae* requires the 2 component amino acids L-cysteine and glycine. L-cysteine is synthesized from L-homocysteine (Hcy), an intermediate of the methyl cycle. Glutathione S-transferases are responsible for catalyzing the conjugation of toxic electrophiles to GSH in preparation for their removal from the cell. *sam2Δ*/sam2*Δ* DEGs are represented by tan (increased expression) and blue (decreased expression) squares. *sam1Δ*/sam1*Δ* DEGs are represented by tan (increased expression) and blue (decreased expression) circles.

The PM plates contain 6 different conditions that could impact ROS levels and/or the glutathione (GSH) biosynthesis pathway, used to respond to ROS (Supplementary Table 5). These conditions can be grouped into 2 categories: drugs that increase ROS levels or decrease the cells’ ability to respond to ROS (sodium selenite, methyl viologen, urea hydrogen peroxide, and thiourea), and drugs that decrease ROS levels or increase the cells’ ability to respond to ROS (sodium thiosulfate). Figure 4a–d shows the growth curves of the wildtype, *sam1Δ/sam1Δ*, and *sam2Δ*/sam2*Δ* strains in response to these compounds. Multiple concentrations were tested in different PM wells and data from a representative well are shown. In general, in the increased ROS/decreased ROS response conditions, the *sam1Δ/sam1Δ* mutant cells exhibit increased adaptation time and equivalent or slightly lowered growth rates and efficiencies compared to wildtype, whereas the *sam2Δ/sam2Δ* mutant cells exhibit either no growth at all (full sensitivity) or similar growth to wildtype (Fig. 4a–c). In the decreased ROS/increased ROS response condition of sodium thiosulfate (Fig. 4d), the *sam1Δ/sam1Δ* mutant cells exhibit increased adaptation time and decreased growth efficiency, while the *sam2Δ/sam2Δ* mutant cells exhibited increased growth rate and decreased adaptation time (Fig. 4d) (for all growth curves, see Supplementary Tables 6 and 7).

Different ROS species have different levels of reactivity, but as a group they have been found to react with and damage most metabolites and macromolecules within the cell (Perrone, Tan, and Dawes 2008). Cells invest in the capacity to neutralize ROS, such as producing reduced GSH, however in the neutralization process, ROS oxidizes GSH to GSSG freezing its anti-oxidative properties (Pizzorno 2014). The GSH-synthesis pathway stems directly from the methyl cycle, where the *SAM1* and *SAM2* genes play essential roles (Fig. 7e) (Prudova *et al*. 2006). Sodium selenite and methyl viologen both increase ROS levels and decrease the cells’ ability to combat ROS, while thiourea only does the latter (Llobell, Fernandez, and López-Barea 1986; Ziegler-Skylakakis *et al*. 2003; Tarze *et al*. 2007; Krieger-Liszkay, Kós, and Hideg 2011; Henderson *et al*. 2014). Conversely, sodium thiosulfate decreases ROS by readily donating electrons to the radical ROS species (Bijarnia *et al*. 2015). In *sam2Δ/sam2Δ* mutant cells, we saw downregulation of *MET6*, *SAM1*, *SAH1*, *CYS3*, *CYS4*, *GSH1*, and *GPX2*, and upregulation of *STR3*, *STR2*, *GRX4*, *GTO1*, and *DUG1* [at cutoffs of *P* ≤ 0.05, −0.58 ≤ log2FC ≥ 0.58, (Supplementary Table 1)]. We did not observe any DEGs in this pathway in the *sam1Δ/sam1Δ* mutant cells beyond *SAM1* itself. Reduced expression of *CYS4*, *CYS3*, and *GSH1* and increased expression of *STR3* and *STR2* in *sam2Δ/sam2Δ* cells (Fig. 4e), points to production of less glutathione overall, such that when the cells are introduced to sodium selenite, methyl viologen, or thiourea, they are more sensitive to the harmful effects due to a reduction of this neutralization mechanism. Sodium thiosulfate donates electrons to radical ROS species, therefore neutralizing them. In this condition, the *sam2Δ/sam2Δ* mutant cells exhibited an increased growth efficiency. This may be explained by an increased expression of *GRX4*, which is responsible for eliminating ROS species by coupling its reduction with the oxidation of GSH to GSSG along with the reduction of a glutaredoxin (Fig. 4e). The *sam2Δ/sam2Δ* mutant cells also express upregulated *GTO1*, part of the family of Gto proteins (composed of Gto1, Gto2, and Gto3), which is a GSH transferase, also responsible for neutralizing ROS species, specifically those that are classified as GS-hydroquinones (Garcerá *et al*. 2006; Belchik and Xun 2011). Therefore, whereas in conditions of increased ROS where these mutants do not appear to generate sufficient glutathione to survive as well as wildtype cells, in this condition where ROS are minimized, the *sam2Δ/sam2Δ* cells appear to be able to combat this reduced load more efficiently through their excess of Grx4 and Gto1. Contrastingly, *sam1Δ/sam1Δ* mutant cells show reduced growth in both sets of conditions, without any pathway DEGs (Fig. 4e), indicating other mechanisms at play in this strain, and that this pathway is not increased in efficiency due to increased AdoMet.

#### D. Lack of *sam2* confers resistance to arginine biosynthesis inhibitors

**Fig. 5.**
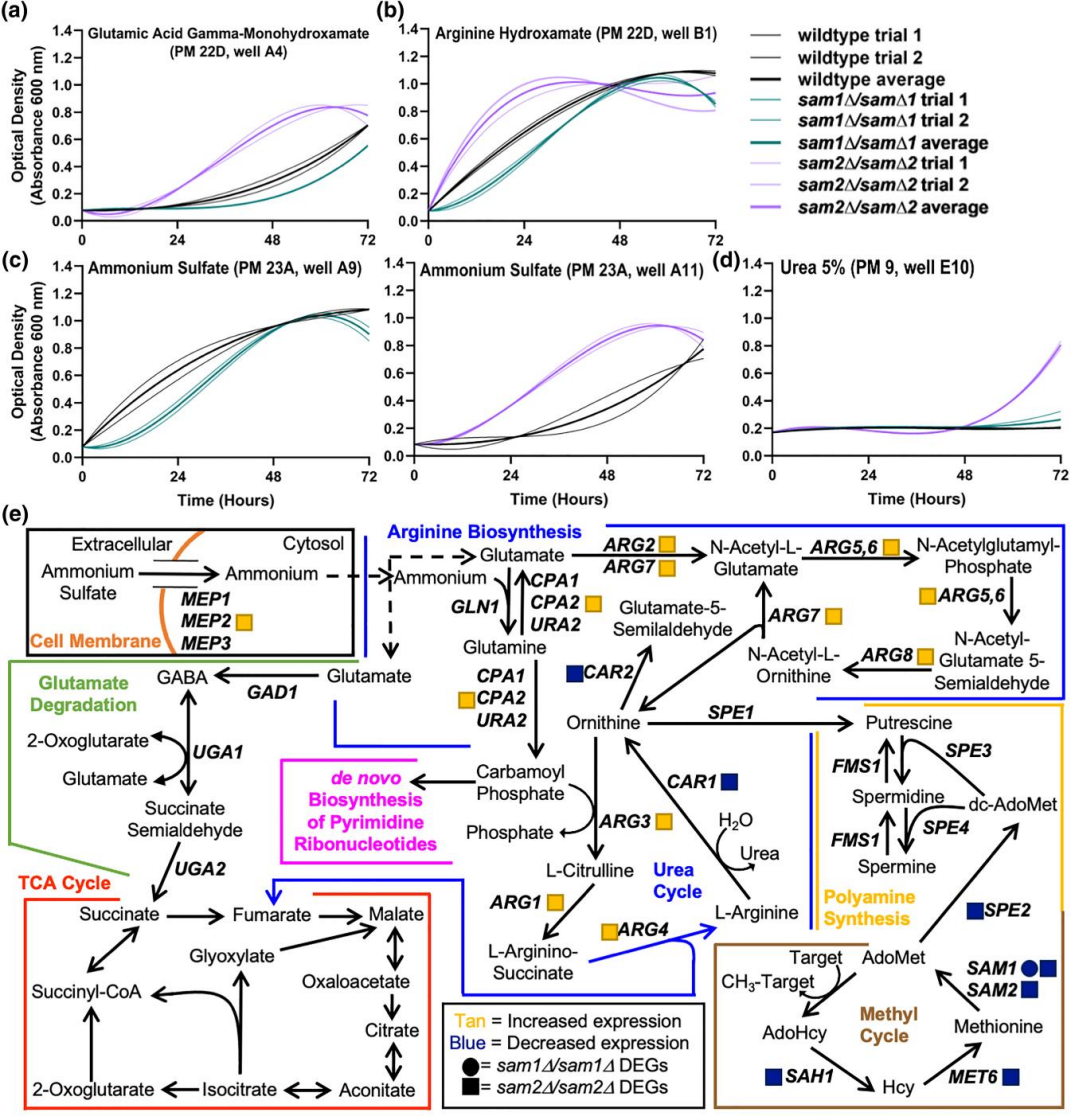
Growth curves of wildtype, *sam1Δ*/sam1*Δ*, and *sam2Δ*/sam2*Δ* cells in the presence of a) L-glutamic acid gamma-monohydroxamate, b) arginine hydroxamate, c) ammonium sulfate, and d) urea 5%, and the effected e) pathways of arginine biosynthesis. Growth curves generated from timepoint data of the Phenotypic Microarray experiments. The independent trials for each strain are shown in a lighter weight line, while the average is shown in the same color in bold. Growth curves are shown for wildtype, *sam1Δ*/sam1*Δ*, and *sam2Δ*/sam2*Δ* cells in a) the highest concentration of L-glutamic acid gamma-monohydroxamate (PM22D, A4) (R-squared Goodness-of-fit values: wildtype trial 1 = 0.9971, wildtype trial 2 = 0.9929, wildtype average = 0.9968, *sam1Δ*/sam1*Δ* trial 1 = 0.9752, *sam1Δ*/sam1*Δ* trial 2 = 0.9740, *sam1Δ*/sam1*Δ* average = 0.9746, *sam2Δ*/sam2*Δ* trial 1 = 0.9976, *sam2Δ*/sam2*Δ* trial 2 = 0.9790, *sam2Δ*/sam2*Δ* average = 0.9907), b) the lowest concentration of arginine hydroxamate (PM22D, B1) (R-squared Goodness-of-fit values: wildtype trial 1 = 0.9973, wildtype trial 2 = 0.9998, wildtype average = 0.9988, *sam1Δ*/sam1*Δ* trial 1 = 0.9852, *sam1Δ*/sam1*Δ* trial 2 = 0.9629, *sam1Δ*/sam1*Δ* average = 0.9744, *sam2Δ*/sam2*Δ* trial 1 = 0.9976, *sam2Δ*/sam2*Δ* trial 2 = 0.9645, *sam2Δ*/sam2*Δ* average = 0.9983), c) the lowest concentration of ammonium sulfate (PM23A, A9) for *sam1Δ*/sam1*Δ* cells (R-squared Goodness-of-fit values: wildtype trial 1 = 0.9916, wildtype trial 2 = 0.9963, wildtype average = 0.9933, *sam1Δ*/sam1*Δ* trial 1 = 0.9936, *sam1Δ*/sam1*Δ* trial 2 = 0.9803, *sam1Δ*/sam1*Δ* average = 0.9894), and the second highest concentration (PM23A, A11) for *sam2Δ*/sam2*Δ* cells (R-squared Goodness-of-fit values: wildtype trial 1 = 0.9888, wildtype trial 2 = 0.9854, wildtype average = 0.9729, *sam2Δ*/sam2*Δ* trial 1 = 0.9980, *sam2Δ*/sam2*Δ* trial 2 = 0.9729, *sam2Δ*/sam2*Δ* average = 0.9977) and d) in 5% urea (PM9, E10) (R-squared Goodness-of-fit values: wildtype no growth = NA, *sam1Δ*/sam1*Δ* trial 1 = 0.9478, *sam1Δ*/sam1*Δ* trial 2 = 0.8779, *sam1Δ*/sam1*Δ* average = 0.9107, *sam2Δ*/sam2*Δ* trial 1 = 0.9018 *sam2Δ*/sam2*Δ* trial 2 = 0.9033, *sam2Δ*/sam2*Δ* average = 0.9025). e) Production of arginine in *S. cerevisiae* begins with synthesis of ornithine from glutamate, followed by conversion to arginine in the urea cycle. In the process, several other substrates are formed that can be used in other biochemical pathways, such as the TCA cycle, polyamine synthesis, and de novo biosynthesis of pyrimidine ribonucleotides. The gene names that encode the proteins involved in the pathway are given by their standard names. *sam2Δ*/sam2*Δ* DEGs are represented by tan (increased expression) and blue (decreased expression) squares. *sam1Δ*/sam1*Δ* DEGs are represented by tan (increased expression) and blue (decreased expression) circles.

We identified 4 different conditions in the PM plates with impacts on arginine biosynthesis and metabolism (Supplementary Table 5), including L-glutamic acid γ-monohydroxamate (Glu-HXM), arginine hydroxamate, ammonium sulfate, and urea 2–7%, and saw altered growth in all (Fig. 5a–d). Multiple concentrations were tested in different PM wells and data from a representative well are shown for wildtype, *sam1Δ/sam1Δ*, and *sam2Δ*/sam2*Δ* strains. In Glu-HXM, arginine hydroxamate, and ammonium sulfate, the *sam1Δ*/sam1*Δ* mutant cells exhibited a decreased growth rate, and had an overall lower growth efficiency, whereas the *sam2Δ*/sam2*Δ* mutant cells had an increased growth rate and increased efficiency (Fig. 5a–d). In 5% urea, the wildtype and *sam1Δ*/sam1*Δ* mutant cells exhibited no significant growth, whereas the *sam2Δ*/sam2*Δ* mutant cells began rapid growth after 48 h (Fig. 5d) (for all growth curves, see Supplementary Tables 6 and 7).

Glu-HXM, a toxic analog of glutamic acid, and arginine hydroxamate, a toxic analog of arginine, are inhibitors of arginine metabolism, while ammonium sulfate and urea are nitrogen sources that are component parts of the pathway (Fig. 5e) (Kisumi *et al*. 1971; Vila *et al*. 1990; Godard *et al*. 2007; Cueto-Rojas *et al*. 2017; Prins and Billerbeck 2021). Arginine metabolism is crucial for cell growth, stress response, metabolic regulation, nitrogen source supplementation, and polyamine synthesis (Takagi 2019). *MEP2*, *CPA2*, *ARG2*, *ARG7*, *ARG5,6*, *ARG8*, *ARG3*, *ARG1*, *ARG4*, and *CAR1* involved in arginine metabolism, and *MET6*, *SAH1*, *SAM1*, *SAM2*, and *SPE2* involved in the methyl cycle and polyamine synthesis are found as DEGs in *sam2Δ/sam2Δ* cells [at cutoffs of *P* ≤ 0.05, −0.58 ≤ log2FC ≥ 0.58, (Supplementary Table 1)]. Notably, almost every gene in the arginine biosynthesis pathway is upregulated. Arginine hydroxamate inhibits arginine biosynthesis, but it has been found that increased concentrations of ornithine and citrulline, produced within the urea cycle, are able to reverse the inhibitory effect (Fig. 5e) (Kisumi *et al*. 1971). We hypothesize that the upregulation of *ARG3* and *ARG7* produces more ornithine and citrulline available to combat the inhibitory effect of arginine hydroxamate, and the upregulation of *ARG4* would produce more arginine to serve as a direct competitor of the toxic analog, allowing the *sam2Δ/sam2Δ* cells to grow better in this condition. Ammonium sulfate is taken up by *S. cerevisiae* as ammonium cations through membrane permeases encoded by genes *MEP1*, *MEP2*, and *MEP3* (Marini *et al*. 1997). Upregulation of *MEP2* allows for increased ammonium uptake for use as a substrate for the synthesis of glutamine from glutamate (Fig. 5e), which can further lead into increased arginine metabolism, TCA cycle and ATP production, and polyamine synthesis due to the upregulation of genes throughout the arginine biosynthesis pathway, together contributing to the increased growth of the *sam2Δ/sam2Δ* cells. As a toxic analog of glutamine, Glu-HXM impacts this pathway (Fig. 5e), but additional studies in *Escherichia coli*, mice, and human cells lines have reported the conversion of Glu-HXM to hydroxylamine (HA) as an active form (Vila *et al*. 1990; Thomasset *et al*. 1992; Boehlein, Schuster, and Richards 1996; Katoh, Hiratake, and Oda 1998). This conversion in *S. cerevisiae* could contribute to the observed increased growth rate and decreased adaptation time as we see direct resistance to HA in our *sam2Δ*/sam2*Δ* cells and the proposed mechanism is discussed at the end of section E below. Urea is a byproduct of conversion of arginine to ornithine by Car1 (Fig. 5e), and is commonly used as a nitrogen source, but in higher concentrations, it can react with ethanol to form ethyl carbamate, a toxic compound that can induce genotoxicity (Monteiro, Trousdale, and Bisson 1989; Hübner *et al*. 1997; Godard *et al*. 2007). *CAR1* and *CAR2* are both involved in arginine degradation and downregulated in *sam2Δ/sam2Δ* cells, which may result in lowered initial concentrations of urea, such that when these cells are exposed to excess urea, they have reduced ethyl carbamate production and survive better than wildtype (Fig. 5e).

#### E. Lack of *sam2* confers resistance to DNA synthesis inhibitors

**Fig. 6.**
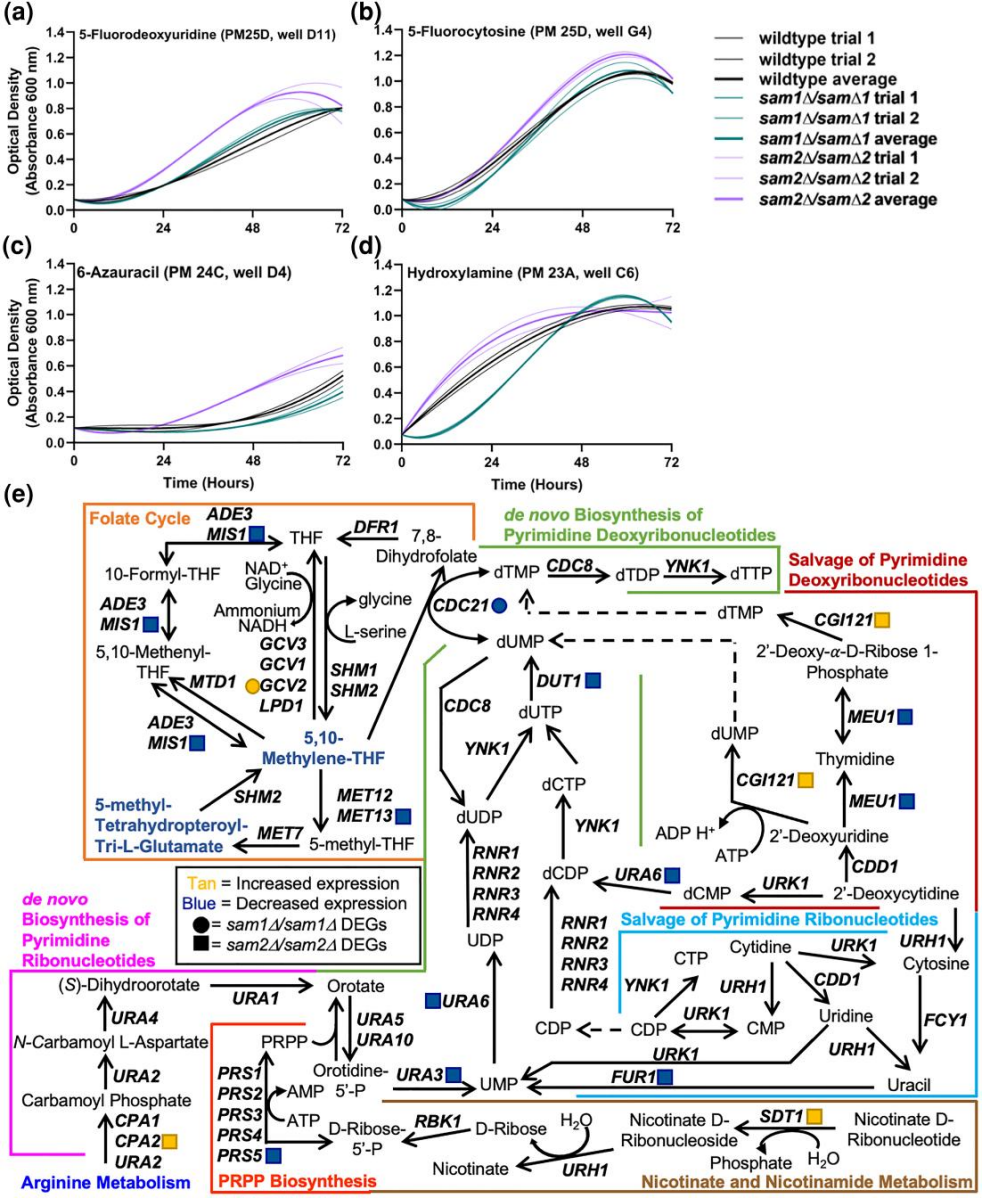
Growth curves of wildtype, *sam1Δ*/sam1*Δ*, and *sam2Δ*/sam2*Δ* cells in the presence of a) 5-fluorodeoxyuridine, b) 5-fluorocytosine, c) 6-azauracil, and d) hydroxylamine, and e) the interconnection between the folate cycle, de novo biosynthesis of pyrimidine nucleotide pathways, salvage pathway of pyrimidine nucleotides, phosphoribosyl diphosphate (PRPP) biosynthesis, and nicotinate and nicotinamide metabolism. Growth curves generated from timepoint data of the Phenotypic Microarray experiments. The independent trials for each strain are shown in a lighter weight line, while the average is shown in the same color in bold. Growth curves are shown for wildtype, *sam1Δ*/sam1*Δ*, and *sam2Δ*/sam2*Δ* cells in a) the second highest concentration of 5-fluorodeoxyuridine (PM25D, D11) (R-squared Goodness-of-fit values: wildtype trial 1 = 0.9950, wildtype trial 2 = 0.9993, wildtype average = 0.9975, *sam1Δ*/sam1*Δ* trial 1 = 0.9910, *sam1Δ*/sam1*Δ* trial 2 = 0.9943, *sam1Δ*/sam1*Δ* average = 0.9927, *sam2Δ*/sam2*Δ* trial 1 = 0.9977, *sam2Δ*/sam2*Δ* trial 2 = 0.9553, *sam2Δ*/sam2*Δ* average = 0.9883), b) in the highest concentration of 5-fluorocytosine (PM25D, G4) (R-squared Goodness-of-fit values: wildtype trial 1 = 0.9966, wildtype trial 2 = 0.9951, wildtype average = 0.9959, *sam1Δ*/sam1*Δ* trial 1 = 0.9814, *sam1Δ*/sam1*Δ* trial 2 = 0.9911, *sam1Δ*/sam1*Δ* average = 0.9882, *sam2Δ*/sam2*Δ* trial 1 = 0.9856, *sam2Δ*/sam2*Δ* trial 2 = 0.9906, *sam2Δ*/sam2*Δ* average = 0.9884), c) in the highest concentration of 6-azauracil (PM24C, D4) (R-squared Goodness-of-fit values: wildtype trial 1 = 0.9594, wildtype trial 2 = 0.9361, wildtype average = 0.9522, *sam1Δ*/sam1*Δ* trial 1 = 0.9080, *sam1Δ*/sam1*Δ* trial 2 = 0.8663, *sam1Δ*/sam1*Δ* average = 0.8897, *sam2Δ*/sam2*Δ* trial 1 = 0.9840, *sam2Δ*/sam2*Δ* trial 2 = 0.9768, *sam2Δ*/sam2*Δ* average = 0.9811), and d) in the third highest concentration of hydroxylamine (PM23A, C6) (R-squared Goodness-of-fit values: wildtype trial 1 = 0.9995, wildtype trial 2 = 0.9987, wildtype average = 0.9991, *sam1Δ*/sam1*Δ* trial 1 = 0.9833, *sam1Δ*/sam1*Δ* trial 2 = 0.9878, *sam1Δ*/sam1*Δ* average = 0.9858, *sam2Δ*/sam2*Δ* trial 1 = 0.9843, *sam2Δ*/sam2*Δ* trial 2 = 0.9740, *sam2Δ*/sam2*Δ* average = 0.9999). e) dNTP pools are maintained through several pathways, including the de novo and salvage pathways of pyrimidines, PRPP biosynthesis, nicotinate and nicotinamide metabolism, and the folate cycle. The gene names that encode the enzymes that function in compound conversion in the pathway are given by their standard names. *sam2Δ*/sam2*Δ* DEGs are represented by tan (increased expression) and blue (decreased expression) squares. *sam1Δ*/sam1*Δ* DEGs are represented by tan (increased expression) and blue (decreased expression) circles. The conversion of dCTP to dUTP is catalyzed by a dCTP deaminase, though the gene encoding this enzyme has not been identified and thus is not labeled in the figure. Through research on homologous genes, we believe that the dCTP deaminase in *S. cerevisiae* could be *DCD1* (Wang and Weiss 1992; Johansson *et al*. 2005), though not confirmed, and not a DEG, and thus not included.

The PM plates contain 7 conditions that could impact DNA synthesis or the folate cycle (Supplementary Table 5), including 5-fluorodeoxyuridine (5-FDU), hydroxylamine (HA), hydroxyurea, 5-fluorocytosine (5-FC), azaserine, 5-fluorouracil, and 6-azauracil (6AU). We observed phenotypic growth differences in our *sam2Δ*/sam2*Δ* strain in 4 of these conditions, 5-FDU, HA, 5-FC, and 6AU. The broad mechanism of action of these drugs is as DNA synthesis inhibitors. Figure 6a–d shows growth curves of the wildtype, *sam1Δ/sam1Δ*, and *sam2Δ*/sam2*Δ* strains; multiple concentrations were tested in different PM wells and data from a representative well are shown. In 5-FDU and 6AU, *sam2Δ*/sam2*Δ* mutant cells exhibited decreased adaptation time and increased growth rate and efficiency (Fig. 6a–c). In 5-FC, the *sam2Δ*/sam2*Δ* mutant cells exhibited increased growth rate, but without a change in adaptation time or growth efficiency (Fig. 6b). In HA, the *sam1Δ*/sam1*Δ* mutant cells exhibited decreased growth rate, whereas the *sam2Δ*/sam2*Δ* mutant cells exhibited a decrease in adaptation time, but otherwise similar growth compared to wildtype (Fig. 6d) (for all growth curves, see Supplementary Tables 6 and 7).

5-Fluorodeoxyuridine, 5-fluorocytosine, 6-azauracil, and hydroxylamine all inhibit DNA synthesis through altering deoxyribonucleoside triphosphate (dNTP) levels, and can therefore impact genome stability (Subramaniam *et al*. 2019). dNTP pools are controlled by 2 major pathways: the de novo biosynthesis pathway and the salvage pathway (Fig. 6e) (Gots 1971). 5-FDU and 5-FC inhibit thymidylate synthase (TS, referred to as TS/Cdc21 moving forward) (Fig. 6e), resulting in depletion of deoxythymidine triphosphate (dTTP) which can inhibit DNA synthesis, imbalance pyrimidine nucleotides, and lead to ineffective DNA repair pathways (Ahmad, Kirk, and Eisenstark 1998). In *sam2Δ*/sam2*Δ* mutant strains, DEGs *CGI121* and *MEU1* in the pyrimidine dNTP salvage pathway would enable the cell to direct 2′-deoxyuridine toward conversion to dUMP, and 2′-deoxy-alpha-D-ribose 1-phosphate toward conversion to dTMP (Fig. 6e), increasing these dTTP precursors to counteract depletion and account for *sam2Δ*/sam2*Δ* resistance. For its effects, 5-FC is first converted into toxic 5-fluorouracil (5-FU) by cytosine deaminase, Fcy1, then 5-FU is converted by uracil phosphoribosyl transferase, Fur1p, to toxic 5-fluorodeoxyuridine 5′-monophosphate (5FdUMP) and 5-fluorouridine 5′-monophosphate (5FUMP). *sam2Δ*/sam2*Δ* cells additionally exhibited decreased expression of *FUR1*, and various mutations in *FUR1* have previously been shown to confer resistance to 5-FC (Jund and Lacroute 1970; Erbs *et al*. 2000). Another portion of the observed resistance could come from increased expression of *CPA2* (Fig. 5e), which has previously been linked to resistance to 5-FU (along with overexpression of *CPA1*) (Carlsson *et al*. 2013).

The uracil analog, 6-azauracil (6AU) also inhibits DNA synthesis, depleting intracellular GTP and UTP concentrations through inhibition of IMP dehydrogenase, Imd2, and orotidylate carboxylase, Ura3, respectively (Fig. 6e) (Bono, Weissman, and Frei 1964). Disruption of *URA3* has been shown to significantly reduce cellular nucleotide levels and inhibit cellular growth (Jones 1992; Zhou *et al*. 2015). Interestingly, in *sam2Δ*/sam2*Δ* cells, there was a significant downregulation of *URA3*, and subsequent genes *URA6* and *DUT1*. Additional research suggests, however, that sensitivity/resistance to 6AU may be more greatly impacted by expression of pyrimidine nucleotidase, an enzyme encoded by *SDT1* (Nakanishi and Sekimizu 2002; Riles *et al*. 2004). 6AU is phosphorylated to its toxic form 6-AzUMP, responsible for competitive inhibition of Ura3 (Fallon *et al*. 1961; Bono, Weissman, and Frei 1964). Sdt1 inactivates 6-AzUMP through dephosphorylation and prevents Ura3 inhibition, ultimately rendering 6AU inert. *SDT1* is overexpressed in *sam2Δ*/sam2*Δ* mutant cells, likely accounting for 6AU resistance and acting upstream of any impacts on Ura3. As well, it is likely that Sdt1 contributes to the hyposensitivity to 5-FC because of its ability to target UMP and its toxic derivatives, 5FUMP, rendering it inert (Nakanishi and Sekimizu 2002).

Research from the 1970s found that HA reacts with the formyl group in 5-formyltetrahyrofolate, and likely other folates, in *S. cerevisiae* (Reeves, Kavanagh, and Whittaker 1992). This reaction forms formoxime, which was shown as an inhibitor of thymidylate synthetase in *E. coli* (Klein *et al*. 1970). In another bacterium, *Mycobacterium bovis*, HA has been shown to target the RNR complex when methylated (Klein *et al*. 1970; Julián *et al*. 2015). While not tested in *S. cerevisiae*, if HA impacts TS/Cdc21, as it does in *E. coli*, the same mechanisms discussed for resistance to 5-FDU and 5-FC likely account for HA resistance. Further, the decreased amount of AdoMet in these cells could limit methylation of HA, if this is a needed step for inhibition of the RNR complex as was seen in *M. bovis*. Conversely, HA was the only one of these conditions where we saw a significant difference in the growth pattern of our *sam1Δ*/sam1*Δ* cells, which could be linked to their increased AdoMet levels. The decreased growth could be due to downregulated *CDC21* expression, which encodes thymidylate synthase, in combination with increased AdoMet/methylation for RNR complex inhibition (Fig. 6e). The lack of sensitivity to 5-FDU or 5-FC points to the *CDC21* downregulation not being significant enough on its own.

#### F. Lack of *sam2* confers resistance to tamoxifen

**Fig. 7.**
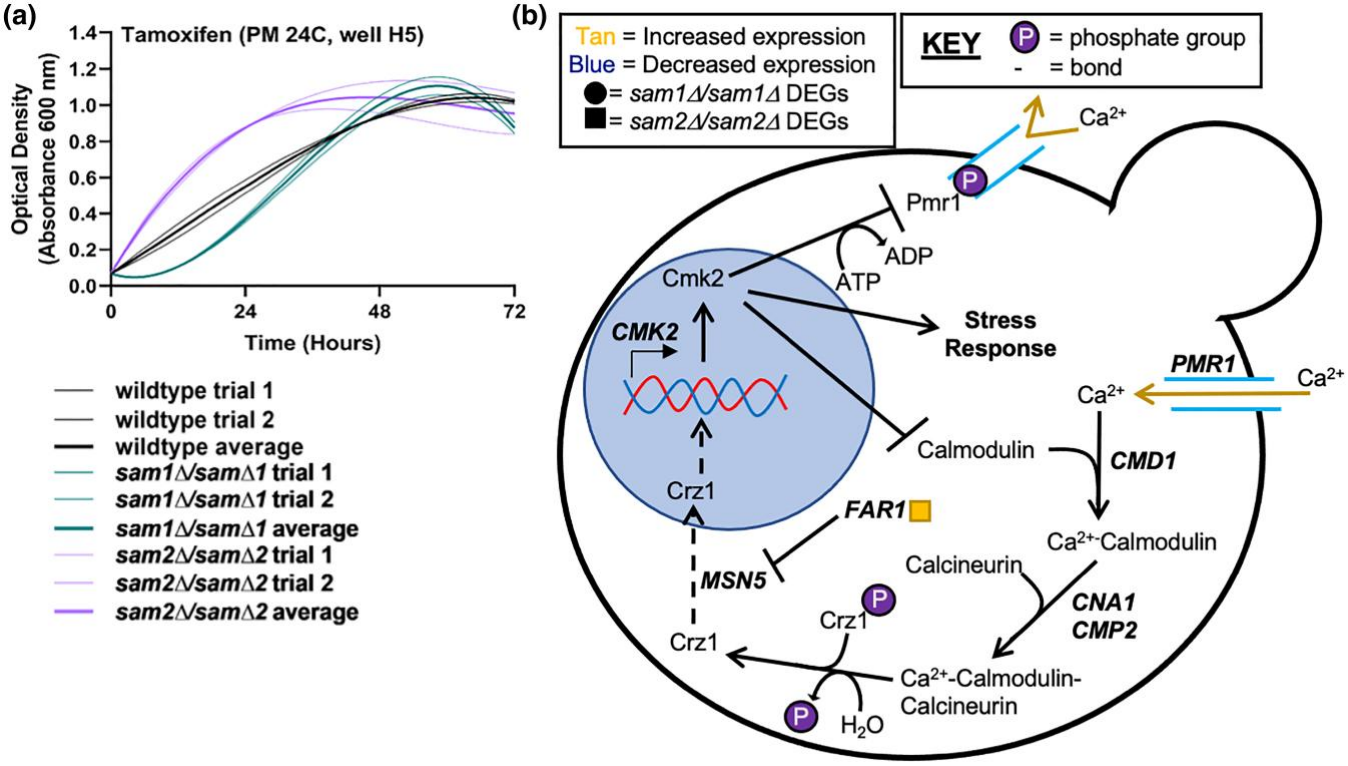
a) Growth curves of wildtype, *sam1Δ*/sam1*Δ*, and *sam2Δ*/sam2*Δ* cells in the presence of tamoxifen, and the impacted b) calcineurin signaling cascade. a) Growth curves generated from timepoint data of the Phenotypic Microarray experiments. The independent trials for each strain are shown in a lighter weight line, while the average is shown in the same color in bold. Growth curves are shown for wildtype, *sam1Δ*/sam1*Δ*, and *sam2Δ*/sam2*Δ* cells in the lowest concentration of tamoxifen (PM24C, H5) (R-squared Goodness-of-fit values: wildtype trial 1 = 0.9985, wildtype trial 2 = 0.9998, wildtype average = 0.9992, *sam1Δ*/sam1*Δ* trial 1 = 0.9757, *sam1Δ*/sam1*Δ* trial 2 = 0.9791, *sam1Δ*/sam1*Δ* average = 0.9775, *sam2Δ*/sam2*Δ* trial 1 = 0.9969, *sam2Δ*/sam2*Δ* trial 2 = 0.9870, *sam2Δ*/sam2*Δ* average = 0.9933). b) Calcium ion signaling in *S. cerevisiae* begins with calcium import by membrane transporters. The calcium ion triggers a signaling cascade with calcineurin and calmodulin, which in response allows for cellular stress response. The gene names that encode the enzymes that can function in compound conversion in the pathway are given by their standard names. *sam2Δ*/sam2*Δ* DEGs are represented by tan (increased expression) and blue (decreased expression) squares. RNA-Sequencing did not reveal any differentially expressed genes within this pathway between *sam1Δ*/sam1*Δ* and wildtype.

The PM plates contain tamoxifen, another known chemotherapeutic. We observed phenotypic growth differences in both of our mutant strains in this condition. Figure 7a shows the growth curves of the wildtype, *sam1Δ/sam1Δ*, and *sam2Δ*/sam2*Δ* strains in response to tamoxifen. *sam2Δ*/sam2*Δ* mutant cells showed an increased growth rate (Fig. 7a), while *sam1Δ*/sam1*Δ* cells showed a slower growth rate initially but caught up to wildtype midway through the experiment (Fig. 7a) (for all growth curves, see Supplementary Tables 6 and 7).

Tamoxifen possess antifungal activity through inhibition of calmodulin binding to calcineurin (Zhang *et al*. 2015; AL-Janabi, Al-Mosawe, and AI-Moswai 2019). Calcineurin and calmodulin play crucial roles in promoting calcium ion signaling pathways in the cell, which assist in regulation of enzymatic activity, metabolic functions, and stress response (Garrett-Engele, Moilanen, and Cyert 1995). Calmodulin, encoded by gene *CMD1*, is activated by calcium ion increases which allow it to bind to calcineurin, encoded by genes *CNA1* and *CMP2* (Fig. 7b) (Sun *et al*. 2019; Creamer 2020). Tamoxifen induces nuclear localization of transcription factor Crz1, leading to transcription of *CMK2* which shuts down Ca^2+^ import and therefore activation of calcineurin/calmodulin (Friedman 1998). Previous research suggests that increased concentrations of calmodulin can overcome the effects of tamoxifen (Dolan *et al*. 2009). The *sam2Δ*/sam2*Δ* mutant cells have increased expression of one gene, *FAR1*, within the calcineurin/calmodulin signaling pathway [at cutoffs of *P* ≤ 0.05, −0.58 ≤ log2FC ≥ 0.58, (Supplementary Table 1)]. *FAR1* is responsible for inhibiting Msn5, which in turn is responsible for nuclear import of proteins, including Crz1 (Fig. 7b) (Hadwiger *et al*. 1989; Görlich *et al*. 1997). When Msn5 is inhibited by Far1, Crz1 does not localize to the nucleus to activate transcription of *CMK2*, less Cmk2 prevents inhibition of Ca^2+^ import and calcineurin/calmodulin activation occurs (Fig. 7b). From the activity of this cascade, we infer that increased Far1 counteracts the effects of tamoxifen, accounting for increased growth rate of *sam2Δ*/sam2*Δ* mutant cells in this condition.

Additional work on tamoxifen resistance in yeast has been carried out in *Schizosaccharomyces pombe*. Two AdoMet-dependent methyltransferases, *EFM6* and *BUD23*, when deleted from *S. pombe*, result in tamoxifen resistance, however the direct mechanisms is unknown (Zhang *et al*. 2015). *S. cerevisiae* homologs of these genes, with the same names, show significant decreases in expression in our *sam2Δ*/sam2*Δ* mutant strain. *EFM6* encodes an AdoMet-dependent lysine methyltransferase, which activates translation elongation factor EF-1 alpha (eEF1A) (Gross and Kinzy 2005; M. E. Munshi *et al*. 2001; Jakobsson *et al*. 2015). *BUD23* encodes a ribosome biogenesis factor and ADMT that methylates 18S rRNA (Sardana, White, and Johnson 2013). Interestingly, Bud23 is known to interact with the rRNA methyltransferase Trm112, also involved in ribosomal biogenesis (Figaro *et al*. 2012), and *TRM112* is significantly downregulated in *sam2Δ*/sam2*Δ* mutant cells. We hypothesize that *sam2Δ*/sam2*Δ* mutant cells are reduced in their ability to carry out these processes via Efm6 and Bud23, contributing to the tamoxifen resistance of these cells.

## Discussion

Methylation is a frequently used regulation mechanism applied to practically all types of cellular macromolecules and used in a wide range of processes and pathways. AdoMet is the main methyl donor in all cell types, and cells therefore have a wide range of AdoMet-dependent methyltransferases to carry out these methylation reactions. Our previous research had identified loss of the 2 AdoMet synthetase encoding genes, *SAM1* and *SAM2*, as resulting in opposite impacts on AdoMet concentration and genome stability. Here, we have presented further characterization of cellular changes due to loss of these 2 genes, through phenotypic profiling and RNA-seq. Of the 1,005 conditions tested via the Phenotypic Microarray, we saw altered growth in ∼10% due to *sam1*-deficiency and ∼15% due to *sam2*-deficiency. The large number of conditions that result in altered growth speaks to the wide array of impacts that altering methyl donor abundance can impart, even when the conditions tested were not specifically selected as targeting known methyl involving pathways. Our RNA-seq experiment was able to capture expression from 7,127 genic regions, and we found 134 DEGs (1.88%) in our *sam1Δ*/sam1*Δ* strain and 876 DEGs (12.29%) in our *sam2Δ*/sam2*Δ* strain. This large percentage of altered gene expression due to *sam2*-deficiency again points to the broad impacts of the gene. Further, *sam2*-deficiency results in ∼50% more PM condition growth differences and approximately 6.5 times as many DEGs, demonstrating that its loss has greater cellular impacts, some of which are through decreased AdoMet concentration. Interestingly, we found 15 AdoMet-dependent methyltransferases downregulated, and 3 upregulated, in our *sam2Δ*/sam2*Δ* cells, as well as 1 upregulated and 1 downregulated non-AdoMet-dependent methyltransferase. This points to a mechanism whereby AdoMet concentration is involved in regulating their transcription, possibly via involvement with binding of activators or repressors or through altered chromatin modifications through reduced methyl donor availability for histone methylation.

Our novel approach combining data sets characterizing the broad changes resulting from loss of these genes allowed us to more widely profile the variety of other impacts that were induced. By utilizing phenotypic profiling and gene expression profiling, we have provided insight that neither data set alone could provide. We have used the information from differential gene expression to show how the phenotypic changes are brought about and have used the phenotypic profiling to show where the gene expression differences are large enough, individually or in combination, to cause altered cellular responses. Some of these changes likely contribute to the genome stability differences previously observed, while others provide insight on additional pathways disrupted by loss of 1 or both AdoMet synthetase genes. Broadly, changes we have characterized are related to (i) AdoMet availability relative to methylation reactions and methyltransferase functionality: azoles and tamoxifen, (ii) AdoMet availability related to methyl cycle components as precursors in synthesis of other important molecules: glutathione and dNTPs, and (iii) impacted genes/pathways with no already defined relationship to methylation, the methyl cycle, or AdoMet itself: cisplatin and arginine metabolism.

### Azoles

Azoles are synthetic organic compounds able to disrupt membrane integrity and ergosterol biosynthesis by targeting lanosterol 14α-demethylase, encoded by *ERG11* (Fig. 2c). Previous studies have elucidated diverse mechanisms of azole resistance including, overexpression/deletion of genes involved in the ergosterol biosynthesis pathway or drug efflux pumps, as well as altered activity of the Set1 histone methyltransferase, or altered vacuolar sequestration (Bhattacharya, Esquivel, and White 2018; Khandelwal *et al*. 2019; Baker *et al*. 2022; Salazar *et al*. 2022). Our novel finding of impacting the ergosterol synthesis pathway through AdoMet and methyltransferase activity, to a large enough extent to result in increased sensitivity (via additional AdoMet) or increased resistance (via decreased AdoMet) has the potential to impact use of azole drugs moving forward. S-Adenosylmethionine is a common over-the-counter nutritional supplement and has also seen increased usage in treatment of a variety of ailments from liver disease to cancers to neurocognitive disorders in recent years (Guo *et al*. 2021; Lu and Mato 2008; Luo *et al*. 2010; Sharma *et al*. 2017). Our studies suggest that addition of AdoMet to fluconazole/azole drug treatment might be effective in increasing sensitivity of other yeasts, like we have shown occurs in *S. cerevisiae*, and is worth further exploration.

### Cisplatin

Cisplatin is most often used to treat cancers of the bladder, testicles, head/neck, lungs, and ovaries (Dasari and Tchounwou 2014). Once in a cell, cisplatin causes damage to DNA (Basu and Krishnamurthy 2010). Consequently, the DNA is unable to be replicated or transcribed, causing cell cycle arrest and apoptosis (Grossmann, Brown, and Moses 1999; Basu and Krishnamurthy 2010). As in *S. cerevisiae*, evidence in human cells shows that cisplatin uptake can occur through facilitated transporters such as the copper transporters (Martinho *et al*. 2019). Previous research has identified several multidrug exporters in human cells that exhibit upregulation in cells resistant to cisplatin (Koshkin *et al*. 2021). We believe our observation that reduced AdoMet levels result in decreased expression of *CTR1*, the main copper transporter, as well as increased expression of drug efflux pumps, with an ultimate impact of reduced sensitivity to cisplatin, warrants further exploration as a cisplatin resistance mechanism in other organisms. Again, the availability of AdoMet as a nutritional supplement and in cancer therapeutics, would allow for exploration of its use as an adjuvant with cisplatin treatment.

### Glutathione

Glutathione (GSH) possesses anti-oxidative properties that are important in protecting cells from oxidative stress. In its reduced form, GSH is a reactant needed to neutralize ROS, such as hydroxyls and superoxides (Whalen and Boyer 1998). ROS can interact directly with nucleic acids in DNA, which has been shown to lead to mutagenesis, and ultimately genome instability. A known connection between GSH and the methyl cycle has been established through the transsulfuration pathway (Fig. 4e) (Sbodio, Snyder, and Paul 2019). Sodium selenite, methyl viologen, and thiourea all have the ability to increase genome instability through ROS. Sodium thiosulfate decreases ROS by readily donating electrons to the radical ROS species, inactivating them and rendering them harmless (Bijarnia *et al*. 2015). Our *sam2Δ*/sam2*Δ* mutants showed better growth in sodium thiosulfate/decreased ROS, while showing sensitivity to sodium selenite. The decreased expression of genes in the methyl cycle and in the synthesis of glutathione from homocysteine (and increased expression of those involved in transforming glutathione back to homocysteine) could point to decreased GSH to combat increased ROS. This pathway could directly be linked to the observed genome instability in these mutants and is under investigation by our group.

### Arginine metabolism

Arginine metabolism is essential to a cell's ability to respond to stress factors, regulate metabolic pathways, synthesize polyamines, and grow and divide (Takagi 2019). The most well understood connection to our *SAM* genes of interest is the involvement in polyamine synthesis which is also linked to the methyl cycle and AdoMet availability. Ornithine serves as the direct link between the urea cycle/arginine biosynthesis and polyamine production (Fig. 5e). It is a precursor to putrescine, an essential precursor to the polyamines spermine and spermidine (Lee *et al*. 2019). Synthesis of polyamines allows for increased DNA repair, stability, and resistance to genotoxic and oxidative damaging substances (Chen *et al*. 2021). For example, evidence suggests that polyamines may promote the Rad51 genome repair complex by exposing damaged DNA to the repair system (Lee *et al*. 2019). Furthermore, raised levels of polyamines in cells have been shown to increase rates of transcription of antioxidant enzymes, thereby lowering ROS (Liu *et al*. 2015). Interestingly, despite an overwhelming increase in expression in most genes in the arginine biosynthesis pathway in our *sam2Δ*/sam2*Δ* mutants, close observation of individual reactions could point to a decrease in available ornithine. Not only is *CAR1* downregulated, responsible for direct conversion of L-arginine to ornithine, but the genes responsible for the reactions that convert ornithine to L-citrulline and further to L-arginino-succinate and L-arginine are all upregulated (*ARG3*, *ARG1*, and *ARG4*, respectively). Decreased available ornithine, in tandem with the previously reported decrease in AdoMet in our *sam2Δ*/sam2*Δ* mutants, could point to decreased components for polyamine synthesis. This could be directly tied to the observed genome instability in these mutants, due to polyamine roles in increased DNA repair, stability and resistance to oxidative damage, and this pathway is under further investigation by our group.

### dNTPs

dNTP levels within the cell must be highly controlled in order to maintain genomic stability (Subramaniam *et al*. 2019). Reduced dNTP pools have been shown to decrease DNA synthesis, thus decreasing the cell's ability to repair DNA and perform recombination (Paulovich *et al*. 1997; Zhao, Muller, and Rothstein 1998; Chabes *et al*. 2003). Research shows that cells can sense dNTP levels and will arrest DNA strand elongation, via halting the replication fork, prior to the complete utilization of remaining dNTPs (Koç *et al*. 2004). Other work has demonstrated a mutator phenotype when dNTP levels exist at higher-than-normal amounts (Chabes *et al*. 2003; Davidson *et al*. 2012; Fleck *et al*. 2013; Pai and Kearsey 2017); attributed to increased dNTPs speeding up S phase, increasing DNA Pol binding/extension from inaccurate primer-template pairing, and reduced proofreading efficiency (Kunkel, Sabatino, and Bambara 1987; Beckman and Loeb 1993; Kunkel and Bebenek 2000; Stodola and Burgers 2016). 5-Fluorodeoxyuridine, 5-fluorocytosine, 6-azauracil, and hydroxylamine all inhibit DNA synthesis. dNTP pools are controlled by de novo biosynthesis and salvage pathways (Fig. 6e). The salvage and de novo pathways interconnect with the folate cycle, and ultimately the methyl cycle, through shared intermediates. Because each of these pathways plays a role in dNTP pool balance, they are likely involved in maintaining genomic stability as well. These pathways also connect to other important cellular pathways, such as arginine metabolism and nicotinamide and nicotinate metabolism (Fig. 5e), which provides insight to how dNTP pools relate to, and are affected by, several other processes within the cell. While the resistance of our *sam2Δ*/sam2*Δ* mutant cells to many DNA damaging agents seems contrary to a link to the increased genome instability at first, we believe the contributing factor is likely in the dNTP pool regulation. We hypothesize the resistance to 5-FDU and 5-FC comes from upregulation of *CGI121* allowing for increased production of dTTP, while 6-AU resistance is from increased expression of *SDT1* encoding pyrimidine nucleotidase which produces ribosides and also dephosphorylates the toxic form of 6-AU inactivating it (Fig. 6e). These gene expression changes could result in altered dNTP levels, and thus be directly linked to the observed genome instability in the *SAM* gene mutants and is under further investigation by our group.

### Tamoxifen

Tamoxifen is a common chemotherapeutic drug, but is also known to possess antifungal activity through inhibition of calmodulin binding to calcineurin (Zhang *et al*. 2015; AL-Janabi, Al-Mosawe, and AI-Moswai 2019). The calcineurin signaling cascade is conserved across all eukaryotes (Aramburu, Heitman, and Crabtree 2004). In human cells, calcium ion signaling not only assists in enzymatic regulation, metabolic functioning, and stress response as seen in *S. cerevisiae* but also aids in cellular proliferation, immune response, apoptosis, and cell differentiation (Park *et al*. 2020). Recent work on additional mechanisms of tamoxifen resistance has been carried out in *S. pombe*, and identified 2 AdoMet-dependent methyltransferase genes, *EFM6* and *BUD23*, that result in tamoxifen resistance when deleted. *S. cerevisiae* homologs for these genes both encode methyltransferases involved in translation, and potentially involved in ribosome production and genome stability as well. The direct mechanism for how decreased functionality of Efm6 and Bud23 lead to tamoxifen resistance has not been uncovered, and while we see the same effect in *S. cerevisiae*, this link has not previously been reported. Both *EFM6* and *BUD23* also have identified homologs in humans (Ferrero *et al*. 2010; Jakobsson, Małecki, and Falnes 2018). In *S. cerevisiae*, eEF1A1 is encoded by *EFM6*, while the closest functional homolog in humans is METTL21B (Malecki *et al*. 2017). Work has shown eEF1A1 to be a novel inhibitor of the tumor-suppressor p53 gene, as well as causing increased resistance to chemotherapeutic drugs (Blanch *et al*. 2013). BUD23, the homolog of the same name in humans, has also been identified in influencing cellular proliferation and tumorigenesis (Bell *et al*. 2020). Bud23 is methylated by Trm112 in *S. cerevisiae*, while Trmt112, the methyltransferase homolog, carries out this reaction in humans. *TRM112* and TRMT112 are AdoMet-dependent methyltransferases, which play a role in ribosome biogenesis and cell proliferation in both humans and *S. cerevisiae*, and have also been identified as being involved in tumor formation in humans (Xu *et al*. 2022). We believe our observation that reduced AdoMet levels result in reduced sensitivity to tamoxifen, should be explored further as a tamoxifen resistance mechanism in other organisms, especially due to the recent increase of AdoMet usage in cancer treatments (Coppola *et al*. 2020; Mosca *et al*. 2020a; Mosca *et al*. 2020b).

### Conclusions/outlook

Our approach and findings on the *SAM* genes in *S. cerevisiae* may provide insight to AdoMet usage and effects of loss in other species as well. AdoMet synthase genes have been studied in other common model organisms, including worms, mice, and flies. Research into the *SAM* genes in other species covers a large spectrum of processes, including embryonic development, extension of lifespan, tumor development, adipocyte maintenance, and cellular regeneration (Menezo *et al*. 1989; Ogawa *et al*. 2016; Swanson *et al*. 2001; Thompson *et al*. 2009; Ehmke *et al*. 2014; Chen *et al*. 2020). Studies of molecular pathways in these organisms overlap with several of the processes we have discussed and align with our findings. *Caenorhabditis elegans* have AdoMet synthase genes *sams-1* through *5*, and work has progressed at separating out expression and function differences in vivo (Walker *et al*. 2011; Ding *et al*. 2018; Godbole *et al*. 2023; Chen *et al*. 2020). This includes findings of opposing phenotypes and distinct gene regulatory effects due to loss of *sams-1* and *sams-4*, and opposite effects on longevity due to knockdown of *sams-1* and *sams-5*, which are in line with the opposite impacts we report here due to loss of *sam1* and *sam2* in yeast (Godbole *et al*. 2023; Chen *et al*. 2020). Studies in *Mus musculus* have linked deletion of *Mat2a* to significant reductions in folates and one-carbon metabolism intermediates (Li *et al*. 2022). MAT2A is homologous to both *SAM1* and *SAM2*, but the largest overall decrease in *SAM* gene expression and lower AdoMet synthetase capacity is seen in our *sam2Δ*/sam2*Δ* cells thus most closely mirroring *Mat2a* deletion. In *sam2Δ*/sam2*Δ* cells, we found multiple decreased DEGs in the folate cycle and one-carbon metabolism (Figs. 5e and 6e), directly in-line with *Mat2a* deletion findings in mice and making an argument for *sam2*-deficiency as a cellular model of *Mat2a* loss. A different mouse study has linked increases in MAT2A protein levels with greatly reduced ROS levels and increased glutathione/oxidized glutathione (GSH/GSSG) ratios pointing to greater antioxidant potential (Chen *et al*. 2022). While we do not have an overexpression/increased expression model, our *sam2Δ*/sam2*Δ* strain represents the opposite scenario and shows the correlating opposite phenotype of increased sensitivity to increased ROS levels and decreased expression leading from the methyl cycle into glutathione biosynthesis (Fig. 4). *Drosophila melanogaster* have a single, essential, AdoMet synthetase gene named *Sam-S*. Knockdown of *Sam-S* has been shown to result in decreased activity of the GSH pathway as well (Liu *et al*. 2020). A different study showed translational machinery modifications that are SAM-dependent involved in control of protein synthesis (Obata *et al*. 2018), which links to our findings of *EFM6* and *BUD23* downregulation in *sam2Δ*/sam2*Δ* cells, where these proteins normally activate protein synthesis through methyltransferase activity related to a translation elongation factor and ribosome biogenesis factor, respectively. Other pathways we have discussed were not immediately found to have been studied in relation to AdoMet in other model systems, but the conservation of findings we see lends support to checking for similar impacts in defining the range of processes impacted by the AdoMet synthetases in higher organisms.

Our novel approach, combining Phenotypic Microarray and RNA-sequencing data sets, has elucidated several very promising avenues for further research. We have identified multiple pathways possessing altered gene expression, warranting further exploration to understand step-by-step how they might contribute to the observed genome instability in our mutants. We have also identified mechanisms of resistance to 3 commonly prescribed drugs due to lowered AdoMet and the resulting altered signaling in *S. cerevisiae*. The pathways these drugs act on have conserved components in *Homo sapiens*, and we suggest investigation of the use of AdoMet supplementation to combat resistance and potentially increase effectiveness of these compounds. Further, the combination of phenotypic profiling and RNA-sequencing methodologies could be executed to learn vast amounts of information about all sorts of organisms and genes.

## Data Availability

Strains are available upon request. The authors affirm that all data necessary for confirming the conclusions of the article are present within the article, figures, tables, supplemental files, and data in GEO. The data discussed in this publication have been deposited in NCBI's Gene Expression Omnibus (Edgar, Domrachev, and Lash 2002) and are accessible through GEO Series accession number GSE249930. RNA-seq data are also available in Supplementary Tables 8 and 9, which contain raw and processed counts, test statistic and *P*-values for *sam1Δ*/sam1*Δ* vs wildtype and *sam2Δ*/sam2*Δ* vs wildtype, respectively. Supplemental data files: Supplementary Table 1 contains data on DEGs. Supplementary Table 2 contains GO Term data. Supplementary Table 3 contains DEG overlaps between strains. Supplementary Table 4 contains summary of PM wells with altered growth. Supplementary Table 5 provides the contents of every PM well. Supplementary Table 6 provides all growth curves for all PM wells for *sam1Δ*/sam1*Δ* vs wildtype. Supplementary Table 7 provides all growth curves for all PM wells for *sam2Δ*/sam2*Δ* vs wildtype. Supplementary Table 8 contains RNA-Seq raw counts, processed counts, and statistical analysis of comparison between *sam1Δ*/sam1*Δ* and wildtype. Supplementary Table 9 contains RNA-Seq raw counts, processed counts, and statistical analysis of comparison between *sam2Δ*/sam2*Δ* and wildtype. Supplementary Fig. 1 contains PCA and Heatmap depictions of RNA-seq data. Supplementary Fig. 2 contains confirmation growth curves for 6 drugs/conditions. Supplemental material included at figshare: https://doi.org/10.25387/g3.24582594.
